# Analytical Performance of ELISA Assays in Urine: One More Bottleneck towards Biomarker Validation and Clinical Implementation

**DOI:** 10.1371/journal.pone.0149471

**Published:** 2016-02-18

**Authors:** Despina Chatziharalambous, Vasiliki Lygirou, Agnieszka Latosinska, Konstantinos Stravodimos, Antonia Vlahou, Vera Jankowski, Jerome Zoidakis

**Affiliations:** 1 Biotechnology Laboratory, Centre of Basic Research, Biomedical Research Foundation of the Academy of Athens, Athens, Greece; 2 Department of Urology, Laikon Hospital, University of Athens, School of Medicine, Athens, Greece; 3 Charité-Universitätsmedizin Berlin, Berlin, Germany; 4 RWTH-Aachen, Institute for Molecular Cardiovascular Research (IMCAR), Aachen, Germany; Eberhard-Karls University, GERMANY

## Abstract

ELISA is the main approach for the sensitive quantification of protein biomarkers in body fluids and is currently employed in clinical laboratories for the measurement of clinical markers. As such, it also constitutes the main methodological approach for biomarker validation and further qualification. For the latter, specific assay performance requirements have to be met, as described in respective guidelines of regulatory agencies. Even though many clinical ELISA assays in serum are regularly used, ELISA clinical applications in urine are significantly less. The scope of our study was to evaluate ELISA assay analytical performance in urine for a series of potential biomarkers for bladder cancer, as a first step towards their large scale clinical validation. Seven biomarkers (Secreted protein acidic and rich in cysteine, Survivin, Slit homolog 2 protein, NRC-Interacting Factor 1, Histone 2B, Proteinase-3 and Profilin-1) previously described in the literature as having differential expression in bladder cancer were included in the study. A total of 11 commercially available ELISA tests for these markers were tested by standard curve analysis, assay reproducibility, linearity and spiking experiments. The results show disappointing performance with coefficients of variation>20% for the vast majority of the tests performed. Only 3 assays (for Secreted protein acidic and rich in cysteine, Survivin and Slit homolog 2 protein) passed the accuracy thresholds and were found suitable for further application in marker quantification. These results collectively reflect the difficulties in developing urine-based ELISA assays of sufficient analytical performance for clinical application, presumably attributed to the urine matrix itself and/or presence of markers in various isoforms.

## Introduction

To establish a protein as a disease biomarker, its accurate, sensitive and reproducible detection and quantification in large numbers of samples representing the biomarker context of use is necessary. The most common methods for protein biomarker validation are affinity-based assays, such as enzyme-linked immunosorbent assays (ELISAs). ELISAs have high sensitivity and reasonable specificity for the detection of protein amounts with concentration ranges of ng/ml to pg/ml in serum. [1] Major limitations of this approach are the restricted number of validated ELISAs for human proteins, the costly and lengthy development of novel assays, and the limited multiplexing due to antibody (Ab) cross-reactivity. [2] These issues hinder the rapid validation of putative biomarkers derived from high-throughput proteomic and genomic studies. [3]

Research based on urine proteomics is crucial for the discovery of disease biomarkers especially of the renal and urogenital systems. In these latter cases, urine is apparently the most appropriate body fluid that can actually be examined for detecting changes related to pathophysiology as it is the filtrate of blood by the kidneys in direct contact with the bladder containing many soluble biomarker proteins. In addition, urine is easily available and can be collected frequently and in a non-invasive way; consisting collectively an appropriate specimen for proteomic biomarker research. [4,5]

Along these lines major efforts have been invested in recent years in biomarker investigations in urine for multiple diseases. [6,7] Bladder cancer (BC) is a major research area where introduction of effective biomarkers is expected to be of major impact on patient management: BC has the highest recurrence rate (approximately 30–70%) among all malignancies and requires extensive patient monitoring for several years. The gold standard for BC initial diagnosis and follow up is cystoscopy (endoscopic examination of the bladder), which is invasive and expensive. Urine cytology which is also used in the clinical setting lacks sensitivity for low grade tumors and is characterized by inter-observer variability. [8] Thus, non-invasive approaches with high sensitivity and specificity for early detection of primary tumors and recurrences are needed. [9,10] An effective BC biomarker could allow reducing the number of unnecessary cystoscopies especially among patients with low risk disease and as a result improve the patients’ quality of life.

As a result of extensive research, several biomarker candidates have been identified following analysis of the urine proteome of bladder cancer patients. [11–15] Nevertheless, despite these efforts, no clinical implementation has been achieved yet, in most part due to lack of appropriate validation studies establishing the biomarker context of use. [16,17] As a first step towards the validation of previously discovered BC biomarker candidates, the objective of this study was to evaluate the analytical performance of ELISA assays in urine. Biomarker candidates include the: NRC-Interacting Factor 1 (NIF-1), Histone 2B (H2B), Profilin-1 (PFN-1), Slit homolog 2 protein (SLIT-2), Proteinase-3 (PR3), and Secreted protein acidic and rich in cysteine (SPARC) and Survivin. [12,18–20] In several cases (NIF-1, H2B, PFN-1) the association of these proteins with BC at the tissue level has been proven [11,12] and initial verification studies in urine have shown discriminatory potential of these marker for bladder cancer detection. [12,18,19] Survivin, has been described in multiple studies as a bladder cancer biomarker, in most cases, based on RT-PCR measurements, [20] but also based on ELISA. [21] Nevertheless, no clear added value for the use of this marker has been demonstrated, in part due to suboptimal assays for its measurement. [20,22]

In this study, extensive analytical validation of commercially available ELISA assays for these markers in urine was performed according to FDA guidelines, as a first step towards the validation of their clinical use. [23] This is particularly interesting since few studies on the analytical performance of ELISA assays in urine are available. [2]

## Materials and Methods

### Urine samples

Urine samples from benign cases and BC patients were collected at the Urology clinic of the Laikon University Hospital, Athens, Greece in accordance to the local ethics regulations. The Ethics committee of Laikon Hospital (protocol number EΣ618) specifically approved the research for this study. In all cases, written consent forms were obtained.

The patients were selected according to the following criteria. Cases had bladder cancer primary tumors; controls suffered from benign urological conditions (hernia, etc).

Clinical data on the urine samples are presented in Table A in S7 File.

The samples were thawed, centrifuged at 2000 rpm for 10 min, and the supernatant was aliquoted to volumes ranging from 0.1 to 1.0 ml. Samples were stored at -20°C and aliquots were thawed for ELISA assays and pH/protein/hematuria determination. Thawed aliquots were not reused. The pH and hematuria of the urine samples was measured by using standard urine analysis strips from EMAPOL and are presented in Table A in S7 File. The protein concentration of the urine samples was measured by the Bradford assay.

### ELISA assays

The following commercially available ELISA kits were tested:

- SPARC: R&D Systems Inc., Minneapolis, MN 55413, USA (Catalogue no. DSP00)
- SLIT-2: Cloud Clone Corp., Houston, TX 77082, USA (Catalogue no. SEA672Hu)
- H2B: US Biological Life Sciences, Swampscott, Massachusetts 01907, USA (Catalogue no. 025705) and Cloud Clone Corp., Houston, TX 77082, USA (Catalogue no. SEA356Hu)
- Survivin: Enzo Life Sciences AG, Postfach CH-4415 Lausen/Switzerland (Catalogue no. ADI-900-111), R&D Systems Inc., Minneapolis, MN 55413, USA (Catalogue no. DSV00)
- PFN-1: USCN LIFE, WUHAN EIAAB SCIENCE CO. LTD, Optics Valley, Wuhan, China (Catalogue no. E2122h); US Biological Life Sciences, Swampscott, Massachussetts 01907, USA (Catalogue no. 027613) and Cloud Clone Corp., Houston, TX 77082, USA (USCN Life Science Inc., Catalogue no. SEC233Hu)
- NIF-1: Cusabio Biotech CO. LTD, Wuhan, Hubei Province 430206, P.R.China (Catalogue no. CSB-EL026683HU) and USCN LIFE, WUHAN EIAAB SCIENCE CO. LTD, Optics Valley, Wuhan, China (Catalogue no. E1019h)
- PR3: Cusabio Biotech CO. LTD, Wuhan, Hubei Province 430206, P.R.China (Catalogue no. CSB- E13058h)

The type of plate reader used was ELx800 (BioTek Instruments).

#### Standard curve validation

Blanks and standards were assayed according to the manufacturer’s instructions in each case. All assays were performed in duplicate and in at least 2 different days. The mean values of Absorbance vs. Concentration were plotted and a 4 Parameter Logistic (4PL) nonlinear regression model) fit was applied (R^2^> 0.95 was acceptable).

#### Recovery

A negative urine sample was spiked with 3 different standards containing high, medium and low concentration of the marker, in 4 replicates each time. The standard protein provided by each ELISA manufacturer was used for the spiking experiments. The % recovery was calculated and the acceptable range was 80 to 120%.

#### Reproducibility

Three urine samples containing high, medium and low concentration of the marker were selected and at least five technical replicates were assayed to calculate the coefficient of variation (CV %) for intra-assay reproducibility. The acceptable range of CV was 0–20%.

The inter-assay reproducibility was evaluated only for the SLIT-2, Survivin, and SPARC since these assays had satisfactory intra-assay reproducibility. Aliquots were used in order to avoid freeze/thaw cycles.

#### Linearity

A urine sample with high marker concentration based on the present study and a published report [11] was selected and serial dilutions (1:2 to 1:32) were performed. Each linearity tests was performed in at least 4 replicates and the experimental versus theoretical concentrations were plotted. The acceptable range was a linear fit with R^2^>0.9 and a slope of 0.9–1.0.

#### Limit of Detection (LOD) and Limit of Quantitation (LOQ)

The LOD was provided by each ELISA kit manufacturer. The LOQ was determined by interpolating the absorbance of the lowest or highest standard on the standard curve.

#### Biomarker evaluation

The t-test was used to evaluate statistical differences between groups (benign controls and tumor stages; tumor grades 1, 2, 3). The effect of hematuria on ELISA results for SPARC, SLIT-2, and Survivin was assessed by the chi-square test.

## Results

Most of the selected proteins had shown discriminatory power as BC biomarkers based on previous studies [11,12] However, no data in urine were available for SLIT-2 and SPARC; thereby these two proteins were initially tested in a small number of BC urine samples and controls (n = 167). In both cases, significantly higher levels of these proteins in BC samples compared to controls were obtained underscoring the need for their further validation. (Figures A, B in S1 File)

**Fig 1:**
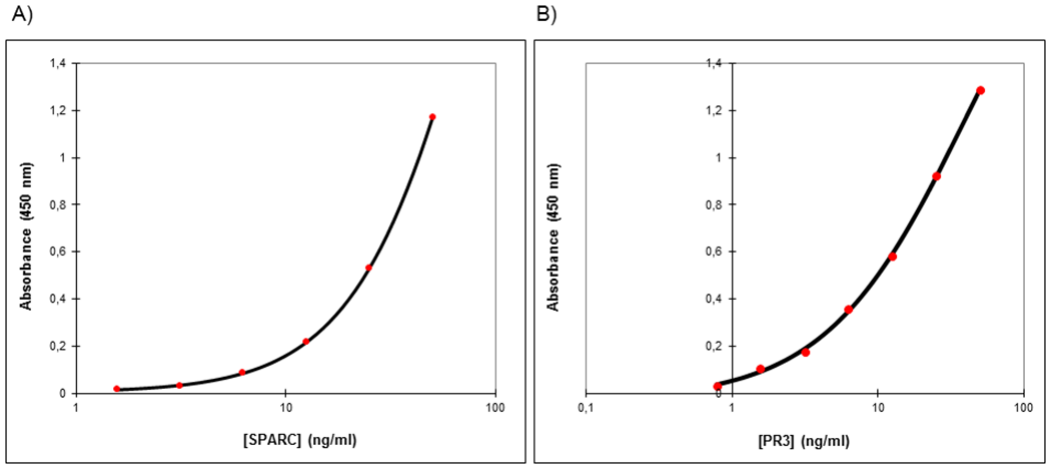
Standard curve validation of A) SPARC (R^2^ = 0.999) and B) PR3 (R^2^ = 0.996).

**Fig 2:**
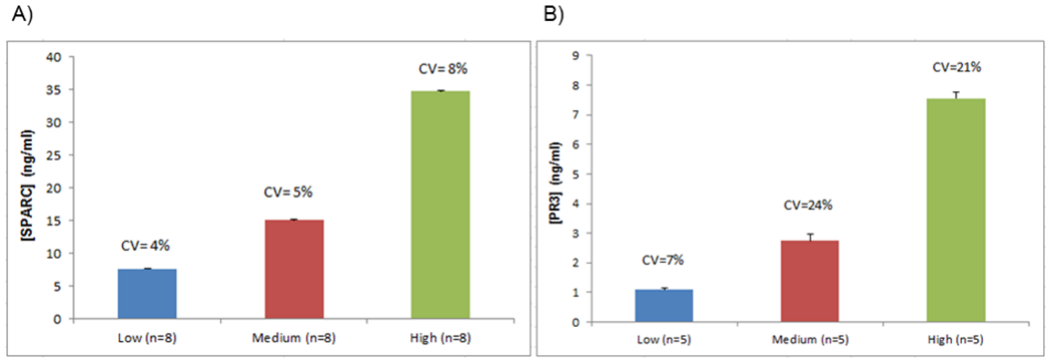
Reproducibility study results of A) SPARC and B) PR3. Three urine samples with low, medium and high concentration were used.

**Fig 3:**
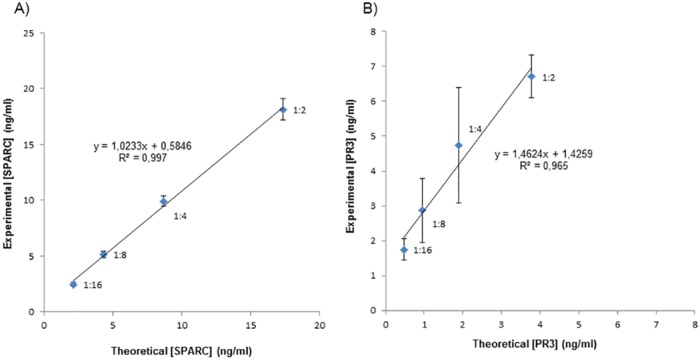
Linearity results of A) SPARC and B) PR3. For each biomarker a high concentration sample was serially diluted and theoretical values were compared to the experimental.

As summarized in Table 1, a total of three ELISA kits targeting respectively SPARC, Survivin and SLIT-2 successfully passed the analytical evaluation tests, whereas a total of 8 assays for NIF-1, PFN-1, PR3 and H2B showed poor analytical performance (Table 1). SPARC (R&D Systems, DSP00) and PR3 (Cusabio Biotech Co. LTD, E13058h) results are presented as examples of successful or poor analytical validation performance respectively (Figs 1–3, Tables 2 and 3), and detailed experimental data for each kit can be found in the supplementary information section. For SPARC, the standards yielded reproducible results and a good fit to the 4 Parameter Logistic (4PL) nonlinear regression model (Fig 1A) Similarly, for PR3, the standards yielded reproducible results and a good fit to the 4PL nonlinear regression model (Fig 1B). In contrast to SPARC, the PR3 assay failed the rest of the analytical performance tests. For SPARC, the % recovery for the medium and high standard was 118% and 108% respectively passing the acceptance threshold (Table 2). Nevertheless, recovery was 136% for the low SPARC levels, reflecting potential inaccuracies in the marker measurements at low concentrations. (Table 2)

**Table 1:** Summary of analytical performance.

Protein	Company	Catalogue number	Analytical performance
**SPARC**	R&D Systems	DSP00	Successful in all assays
**SLIT-2**	Cloud Clone Corp.	SEA672Hu	Failed in linearity assay
**H2B**	US Biological Life Sciences	25705	Failed in recovery and reproducibility assays (linearity not possible)
	Cloud Clone Corp.	SEA356Hu	Failed in recovery and reproducibility assays (linearity not possible)
**SURVIVIN**	Enzo Life Sciences	ADI-900-111	Failed in recovery, reproducibility and linearity assays
	R&D Systems	DSV00	Successful in all assays (linearity not possible)
**PFN-1**	USCN LIFE	E2122h	Failed in recovery, reproducibility and linearity assays
	US Biological Life Sciences	27613	Failed in recovery, reproducibility and linearity assays
	Cloud Clone Corp.	SEC233Hu	Failed in reproducibility and linearity assays
**NIF-1**	CUSABIO	EL026683HU	Failed in recovery, reproducibility and linearity assays
	USCN LIFE	E1019h	Failed in recovery and linearity assays
**PROTEINASE 3**	CUSABIO	E13058h	Failed in recovery, reproducibility and linearity assays

**Table 2:** Recovery study results of SPARC. Negative urine samples were spiked with low, medium and high concentration of standard.

Negative + 3.13 ng/ml SPARC (n = 8)	Mean [SPARC] (ng/ml)	2.14
	Expected [SPARC] (ng/ml)	1.57
	% Recovery	***136%***
Negative + 12.5 ng/ml SPARC (n = 8)	Mean [SPARC] (ng/ml)	7.4
	Expected [SPARC] (ng/ml)	6.25
	% Recovery	***118%***
Negative + 50 ng/ml SPARC (n = 8)	Mean [SPARC] (ng/ml)	27
	Expected [SPARC] (ng/ml)	25
	% Recovery	***108%***

**Table 3:** Recovery study results of PR3. Negative urine samples were spiked with low, medium and high concentration of standard.

Negative + 1.56 ng/ml PR3 (n = 4)	Mean [PR3] (ng/ml)	2.10
	Expected [PR3] (ng/ml)	0.78
	% Recovery	***269%***
Negative + 6.25 ng/ml PR3 (n = 4)	Mean [PR3] (ng/ml)	4.21
	Expected [PR3] (ng/ml)	3.13
	% Recovery	***135%***
Negative + 25 ng/ml PR3 (n = 4)	Mean [PR3] (ng/ml)	15.74
	Expected [PR3] (ng/ml)	12.50
	% Recovery	***126%***

In contrast, for PR3, the % recovery for the low, medium, and high standards was 269%, 135%, and 126% respectively (Table 3) clearly exceeding the allowed acceptable recovery range.

When tested for reproducibility in measurement using high, medium and low biomarker concentrations, as described in Materials and Methods section satisfactory CVs were obtained for SPARC. For the low [SPARC] sample a CV of 4%, for the medium [SPARC] sample a CV of 5% and for the high [SPARC] sample, a CV of 8% was obtained. (Fig 2A)

For PR3, the CV% was above the acceptable 20% limit for the medium and high [PR3] samples (24% and 21% respectively). The low [PR3] sample had a satisfactory CV (7%). (Fig 2B)

When further tested for linearity the performance of the ELISA assay for SPARC was excellent from dilution 1:2 up to 1:16 (R^2^ = 0.997 and a slope of 1.023). (Fig 3A) The respective values for PR3 were R^2^ = 0.965 which is acceptable, and a slope of 1.46 which is not acceptable. (Fig 3B)

The majority of the remaining ELISA kits, even though successful for the standard curve validation, failed either in reproducibility, or in recovery and linearity studies. For example, in the spiking experiments of pure recombinant standards to negative urine samples, extremely low (e.g. PFN-1 Elisa kit by US Biological, Figure G in S2 File) or high (e.g. NIF-1 Elisa kit by CUSABIO, Figure I in S2 File) % recoveries were obtained. It is important to note that particularly poor results were obtained in the linearity test for most of the assays (Figures A-G in S4 File).

The inter-assay reproducibility was evaluated for SLIT-2, Survivin, and SPARC since only these assays had satisfactory intra-assay reproducibility. The CVs of the inter-assay reproducibility for these 3 ELISA kits are reported in Table A in S9 File. For SPARC the CVs of the urine samples with low, medium and high concentration were 29%, 9% and 34% respectively. For SLIT-2 the CVs of the urine samples with low, medium and high concentration were 43%, 34% and 11% respectively. For Survivin only the CV of a low concentration urine sample could be assessed and was determined to be 41% (the available clinical urine samples were either negative or had low Survivin concentration). Aliquots were used in order to avoid freeze/thaw cycles.

The LOD and LOQ for each ELISA kit are listed in Table A in S10 File.

Hemoglobin released from erythrocyte lysis was measured by standard urine analysis strips (EMAPOL) and its effect on the ELISA assay is reported. (Figure A in S5 File, Table A in S11 File, Table A in S12 File, Table A in S13 File). Hematuria affected significantly only the Survivin ELISA assay as it is was determined by the chi-square statistical test.

The values of the SPARC and SLIT-2 ELISA kits and their dependence on tumor grade are presented. (Figures A, B in S6 File) There is a gradual increase in the SLIT-2 values when tumor grade increases but there is no statistically significant difference. In the case of SPARC Grade 2 tumors have higher mean value compared to G1 and G3 without any statistically significant difference. In both SPARC and SLIT-2 data the standard deviation is very high.

However given the limited number of samples analyzed, a more comprehensive multi-center study is under way for evaluating the effect of tumor grade on SPARC and SLIT-2 ELISA results.

## Discussion

Recently, several urine-based bladder tumor markers have been evaluated and are implicated in non-invasive clinical tests for BC detection. [24,25] The commercially available ELISA assays include BTA, nuclear matrix protein 22, AccuDx, and UBC. Unfortunately these ELISA urine biomarkers do not have better performance than cystoscopy and are significantly affected by the presence of hematuria. There is no clearly demonstrated added value for using them in initial diagnosis or patient monitoring. [17]

Urine complexity hinders the development of methods for precise and reproducible protein quantitation. [5] Urine contains more than 1,500 proteins, the majority of which are extracellular and membrane bound along with cells and cellular debris, inorganic ions (K^+^, Na^+^, Cl^−^ and Ca^2+^) and organic molecules such as creatinine, urea, and uric acid. All these substances can hinder the efficient binding of a protein to its corresponding antibody used in an ELISA assay. [12,26] Variability of urine matrix components such as electrolytes or pH can also have an effect on antibody binding and therefore on the performance of the immunoassay. [27] In the case of multiplex bead array assays, to compensate for the impact of matrix effects on biological fluids, manufacturers have developed standard sample diluents for serum, plasma, cultured cells. For urine, a diluent of phosphate buffered saline is recommended for use; however this does not resolve the issue of variability of urine matrix components as the measurements appear less stable compared to those in serum and plasma. [28] To our knowledge the only urinary protein measured by ELISA in clinical laboratories is albumin. [4]

Nevertheless even in the case of albumin measurements multiple limitations have been identified, mostly related to the presence of the protein in multiple isoforms. Many of these forms are considered different to those in plasma. Currently, a reference standard material for urine albumin is not available therefore serum albumin is used for calibration in urine assays. In healthy individuals serum albumin, when filtered and excreted in urine, is composed of a minor amount of intact protein (~4%) and a large amount of albumin fragments with MW in the 1–15 kDa range (~96%). [29] However, it was shown that diabetic nephropathy gradually increases the percentage of intact albumin in urine up to 35% in severe cases. [30] Conventional ELISA assays can detect only certain forms of albumin and the antibodies used fail to bind efficiently to many isoforms. [31] Despite these limitations Albumin ELISA is routinely used in clinical laboratories mainly for diagnosis of kidney diseases. [32]

In light of these findings for one of the most abundant urinary proteins it is imperative to evaluate the analytical performance of ELISA kits for the detection of candidate biomarkers in urine. The FDA guidelines for Bioanalytical Method Validation were followed. [23] Unfortunately, most ELISA assays used in this study did not pass these strict analytical criteria. Some explanations for these disappointing results are presented along with a comparison to previous urinary ELISA analytical performance studies.

Low recovery may be due to interference of antigen recognition caused by substances present in urine (salts, organic molecules, etc.). High recovery may be due to non-specific binding of proteins to the antibody immobilized on the ELISA plate. In a study by Taylor et al., in order to determine the degree of matrix interference in protein measurement in urine, known concentrations of 5 proteins (IL-6, IL-8, MCP1, MP1a and TNFα) were spiked in urine samples of 4 kidney disease patients and assayed 4 times each. High variability was observed in protein recovery in the urine samples even between assays indicating that matrix components differ among urine samples and also highlighting their ability to variably interfere in accurate protein measurement. [28]

Inter-assay reproducibility results were not acceptable (high CVs) for the three kits that yielded satisfactory intra-assay reproducibility (SPARC from R&D Systems, SLIT-2 from Cloud-Clone Corp. and Survivin from R&D Systems) (Table A in S9 File)

The failure of the linearity test is the major deficiency of most ELISA kits analyzed. A possible explanation of this deficiency is the fact that in urine proteins exist in multiple forms with different affinities for the ELISA antibodies. As it was determined for Albumin, urinary proteins are not present only as full length polypeptides but also as numerous low MW peptides and exhibit unique post-translational modifications (PTMs) different from those in plasma. [30,31,33] It is possible that some of these forms have higher Kd than the full length and do not bind to the Ab upon dilution resulting in lower signal. (Figures D, G in S3 File) Moreover, the linearity of the assay can be affected by the dilution of interfering salts and organic molecules. As a result protein-Ab binding is enhanced and a higher signal is obtained (Figures A, F in S3 File). In the case of Survivin and SLIT-2, urine sample desalting was performed before ELISA analysis. Unfortunately the desalting did not increase signal intensity and thus did not improve assay performance (data not shown). For the two H2B Elisa kits and the Survivin Elisa kit from R&D Systems the linearity tests could not be performed due to the unavailability of high concentration samples and the minimum detectable dose of each kit.

The poor performance of ELISA assays in urine presented in this study is not a unique occurrence. A comprehensive evaluation of the analytical performance of ELISA assays for Neutrophil gelatinase-associated lipocalin (NGAL) yielded poor results for recovery and linearity. These findings indicated the presence of variability in urinary immunoassay performance that needs to be taken into consideration in clinical sample analysis. [34]

The performance of SLIT-2 and SPARC in detecting BC recurrence and/or progression will be assessed in the context of a large clinical study involving prospectively collected samples. The effect of confounders, such as hematuria, on the ELISA assays and the diagnostic performance of SPARC and SLIT-2 individually or in combination will be evaluated.

The shortcomings of the assays presented in this article reflect the difficulties on developing robust ELISA in urine for clinical applications. An alternative to ELISA assays would be to develop MRM (Multiple Reaction Monitoring) methods for determining biomarker concentration in urine. Beasley-Green et al., employed isotope dilution–mass spectrometry (ID–MS) and multiple reaction monitoring (MRM) as a reference method to measure full-length albumin and its fragments in urine. The assay showed outstanding specificity, reproducibility and sensitivity. Thus, MRM has the potential to be applied in the clinical setting for biomarker measurements. [35]

## Supporting Information

S1 File Preliminary clinical data of SLIT-2 (Figure A), SPARC (Figure B)(*p≤0.05). (DOCX)

S2 File Standard curve validation of SLIT-2 (Cloud-Clone Corp. USCN Life Science Inc., SEA672Hu) (Figure A) H2B (US Biological Life Sciences, 025705) (Figure B) H2B (Cloud-Clone Corp. USCN Life Science Inc., SEA356Hu) (Figure C) Survivin (Enzo Life Sciences, ADI-900-111) (Figure D) Survivin (R&D Systems Inc., DSV00) (Figure E) PFN-1 (USCN Life, WUHAN EIAAB SCIENCE CO. LTD, E2122h) (Figure F) PFN-1 (US Biological Life Sciences, 027613) (Figure G) PFN-1 (Cloud-Clone Corp., USCN Life Science Inc., SEC233Hu) (Figure H) NIF-1 (Cusabio Biotech CO. LTD, CSB-EL026683HU) (Figure I) NIF-1 (USCN Life, WUHAN EIAAB SCIENCE CO. LTD, E1019h) (Figure J). (DOCX)

S3 File Reproducibility study results of SLIT-2 (Cloud-Clone Corp. USCN Life Science Inc., SEA672Hu) (Figure A) H2B (US Biological Life Sciences, 025705) (Figure B) H2B (Cloud-Clone Corp. USCN Life Science Inc., SEA356Hu) (Figure C) Survivin (Enzo Life Sciences, ADI-900-111) (Figure D) Survivin (R&D Systems Inc., DSV00) (Figure E) PFN-1 (USCN Life, WUHAN EIAAB SCIENCE CO. LTD, E2122h) (Figure F) PFN-1 (US Biological Life Sciences, 027613) (Figure G) PFN-1 (Cloud-Clone Corp., USCN Life Science Inc., SEC233Hu) (Figure H) NIF-1 (Cusabio Biotech CO. LTD, CSB-EL026683HU) (Figure I) NIF-1 (USCN Life, WUHAN EIAAB SCIENCE CO. LTD, E1019h) (Figure J). (DOCX)

S4 File Linearity results of SLIT-2 (Cloud-Clone Corp. USCN Life Science Inc., SEA672Hu) (Figure A) Survivin (Enzo Life Sciences, ADI-900-111) (Figure B) PFN-1 (USCN Life, WUHAN EIAAB SCIENCE CO. LTD, E2122h) (Figure C) PFN-1 (US Biological Life Sciences, 027613) (Figure D) PFN-1 (Cloud-Clone Corp., USCN Life Science Inc., SEC233Hu) (Figure E) NIF-1 (Cusabio Biotech CO. LTD, CSB-EL026683HU) (Figure F) NIF-1 (USCN Life, WUHAN EIAAB SCIENCE CO. LTD, E1019h) (Figure G). (DOCX)

S5 File Bar plot results of SPARC (R&D Systems), SLIT-2 (Cloud Clone Corp.) and SURVIVIN (R&D Systems) ELISA (positive/negative) relative to the presence/absence of hematuria (* p<0.05). (Figure A). (DOCX)

S6 File ELISA results of SPARC (Figure A) and SLIT-2 (Figure B) relative to cancer grade. (DOCX)

S7 File Urine strip analysis, ELISA results, and clinical data for urine samples. (Table A). (DOCX)

S8 File Recovery study results of SLIT-2 (Cloud-Clone Corp. USCN Life Science Inc., SEA672Hu) (Table A) H2B (US Biological Life Sciences, 025705) (Table B) H2B (Cloud-Clone Corp. USCN Life Science Inc., SEA356Hu) (Table C) Survivin (Enzo Life Sciences, ADI-900-111) (Table D) Survivin (R&D Systems Inc., DSV00) (Table E) PFN-1 (USCN Life, WUHAN EIAAB SCIENCE CO. LTD, E2122h) (Table F) PFN-1 (US Biological Life Sciences, 027613) (Table G) PFN-1 (Cloud-Clone Corp., USCN Life Science Inc., SEC233Hu) (Table H) NIF-1 (Cusabio Biotech CO. LTD, CSB-EL026683HU) (Table I) NIF-1 (USCN Life, WUHAN EIAAB SCIENCE CO. LTD, E1019h) (Table J). (DOCX)

S9 File Inter-assay reproducibility results for SPARC, SLIT-2 and SURVIVIN. (Table A). (DOCX)

S10 File LOD and LOQ information for all the ELISA kits. (Table A). (DOCX)

S11 File Chi-square test results of SPARC and hematuria. (Table A). (DOCX)

S12 File Chi-square test results of SLIT-2 and hematuria. (Table A). (DOCX)

S13 File Chi-square test results of SURVIVIN and hematuria. (Table A). (DOCX)
