# Supplementary material for: Analytical Performance of ELISA Assays in Urine: One More Bottleneck towards Biomarker Validation and Clinical Implementation
Source: PLoS One. 2016 Feb 18;11(2):e0149471. doi: 10.1371/journal.pone.0149471 (PMC4758723; doi:10.1371/journal.pone.0149471)
Supplement: S1 File — (DOCX) [file pone.0149471.s001.docx]

**ELISA data on clinical samples**

For SLIT-2, the majority of the benign (48/52), Ta (41/46), T1 (34/36) and T2+ (28/29) samples were positive. Also, theT2+ samples showed a statistical difference compared to Benign and Ta groups (p-value<0.05). For SPARC, very few benign (4/20), Ta (2/21) and T1 (4/20) samples were positive compared to the T2+ positive samples (9/21). Furthermore, the T2+ samples showed a statistical difference compared to the other groups (*p-value<0.05). (**Figs A, B**)

**
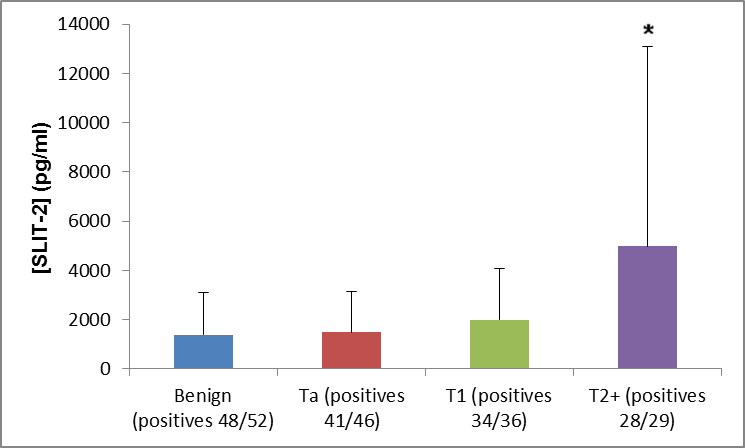
**

**Figure A. ELISA data in urine samples (Benign controls and BC cases Ta, T1, T2+) for SLIT-2. (*p≤0.05)**

**Figure B. ELISA data in urine samples (Benign controls and BC cases Ta, T1, T2+) for SPARC. (*p≤0.05)**
