# Supplementary material for: Analytical Performance of ELISA Assays in Urine: One More Bottleneck towards Biomarker Validation and Clinical Implementation
Source: PLoS One. 2016 Feb 18;11(2):e0149471. doi: 10.1371/journal.pone.0149471 (PMC4758723; doi:10.1371/journal.pone.0149471)
Supplement: S2 File — (DOCX) [file pone.0149471.s002.docx]

**Standard curve validation**

For SPARC (R&D Systems Inc., DSP00), a standard curve was obtained from 1 blank and 6 standards (1.56 to 50ng/ml) that were analyzed in duplicates on 3 different days. The 4PL fit gave a R^2^=0.999. (**Fig.1A**)

For PR3 (Cusabio Biotech CO. LTD, CSB-E13058h), a standard curve was obtained from 1 blank and 7 standards (0.78 to 50ng/ml) that were analyzed in duplicates on 2 different days. The 4PL fit gave a R^2^=0.996. (**Fig.1B**)

For SLIT-2 (Cloud-Clone Corp. USCN Life Science Inc., SEA672Hu), a standard curve was obtained from 1 blank and 7 standards (78 to 5000pg/ml) that were analyzed in duplicates on 3 different days. The 4PL fit gave a R²=0.9997. (**Figure A**)


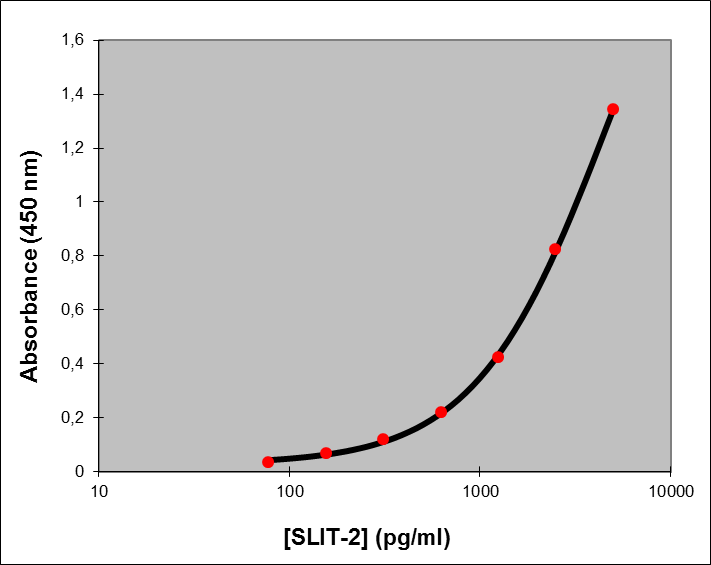


**Figure A. Standard curve validation of SLIT-2.**

For H2B (US Biological Life Sciences, 025705), a standard curve was obtained by 1 blank and 7 standards (3.12 to 200ng/ml) that were analyzed in duplicates on 2 different days. The 4PL fit gave a R²=0.9996. (**Figure B**)


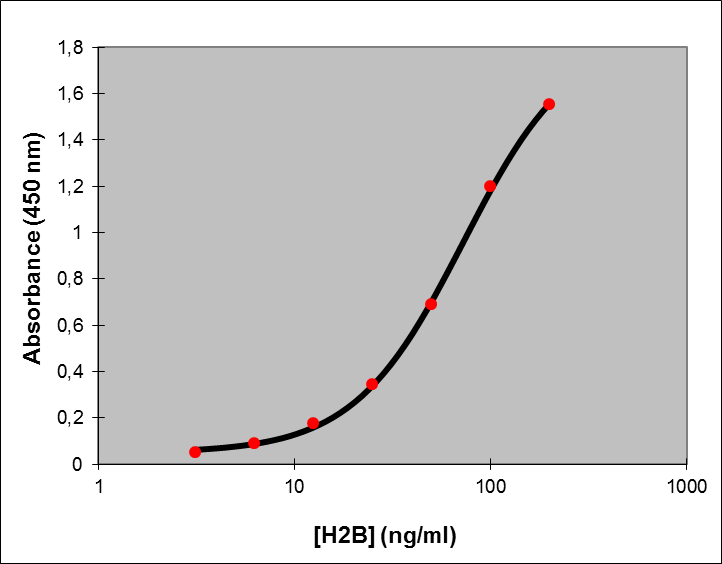


**Figure B. Standard curve validation of H2B.**

For H2B (Cloud-Clone Corp. USCN Life Science Inc., SEA356Hu), a standard curve was obtained from 1 blank and 7 standards (3.12 to 200ng/ml) that were analyzed on duplicates in 2 different days. The 4PL fit gave a R²=0.9996. (**Figure C**)

**
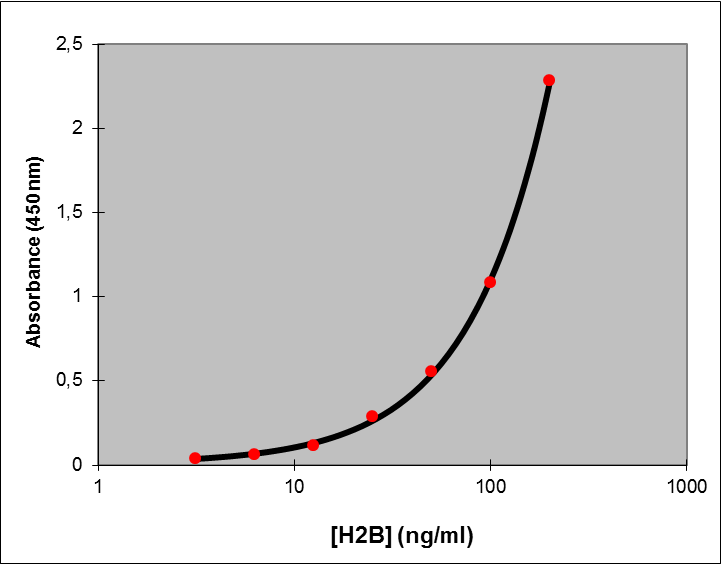
**

**Figure C.** **Standard curve validation of H2B.**

For Survivin (Enzo Life Sciences, ADI-900-111), a standard curve was obtained from1 blank and 5 standards (31.25 to 500pg/ml) that were analyzed in duplicates on 3 different days. The 4PL fit gave a R²=0.9995. (**Figure D**)

**
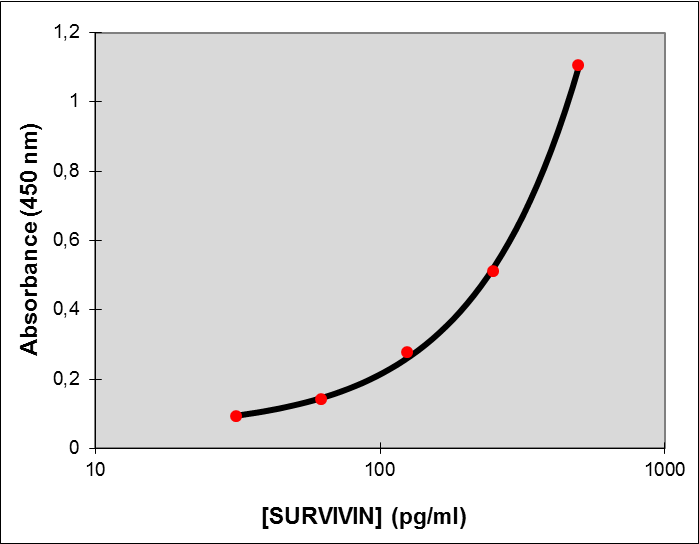
**

**Figure D. Standard curve validation of SURVIVIN.**

For Survivin (R&D Systems Inc., DSV00), a standard curve was obtained from 1 blank and 7 standards (31.2 to 2000pg/ml) that were analyzed in duplicates on 3 different days. The 4PL fit gave a R²=0.9995. (**Figure E**)

**
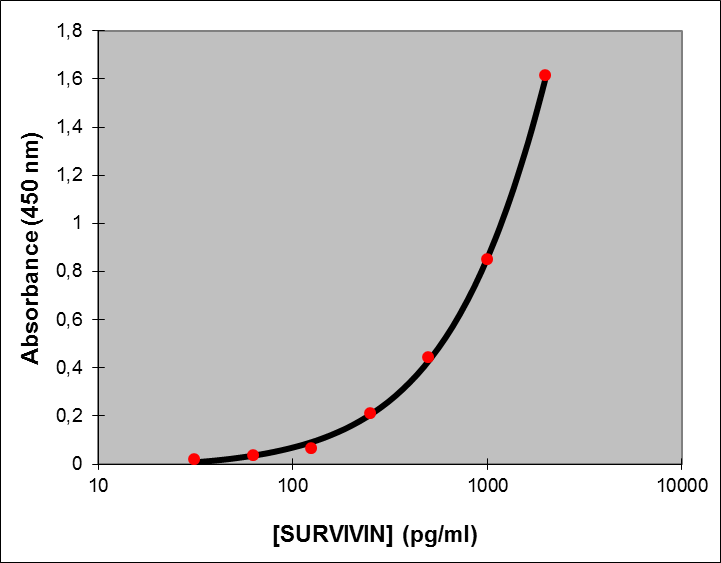
**

**Figure E.** **Standard curve validation of SURVIVIN.**

For PFN-1 (USCN Life, WUHAN EIAAB SCIENCE CO. LTD, E2122h), a standard curve was obtained from 1 blank and 7 standards (78 to 5000pg/ml) were analyzed in duplicates on 2 different days. The 4PL fit gave a R²=0.9993. (**Figure F**)

**
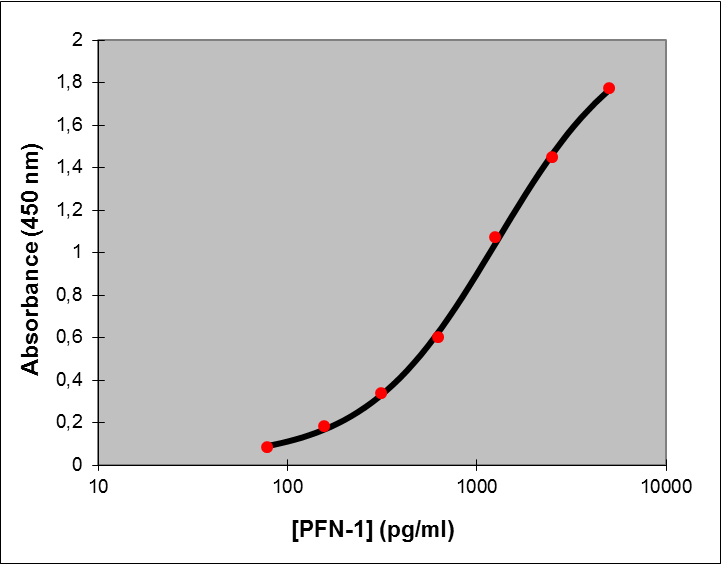
**

**Figure F.** **Standard curve validation of PFN-1.**

For PFN-1 (US Biological Life Sciences, 027613), a standard curve was obtained from 1 blanks and 7 standards (78 to 5000pg/ml) that were analyzed in duplicates on 2 different days. The 4PL fit gave a R²=0.9999. (**Figure G**)

**
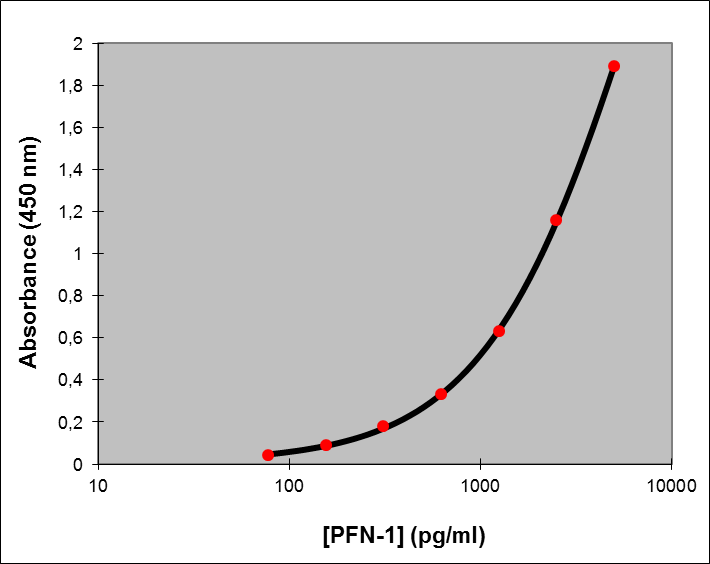
**

**Figure G.** **Standard curve validation of PFN-1.**

For PFN-1 (Cloud-Clone Corp., USCN Life Science Inc., SEC233Hu), a standard curve was obtained from1 blank and 7 standards (78 to 5000pg/ml)that were analyzed in duplicates on 2 different days. The 4PL fit gave a R²=0.9934. (**Figure H**)

**
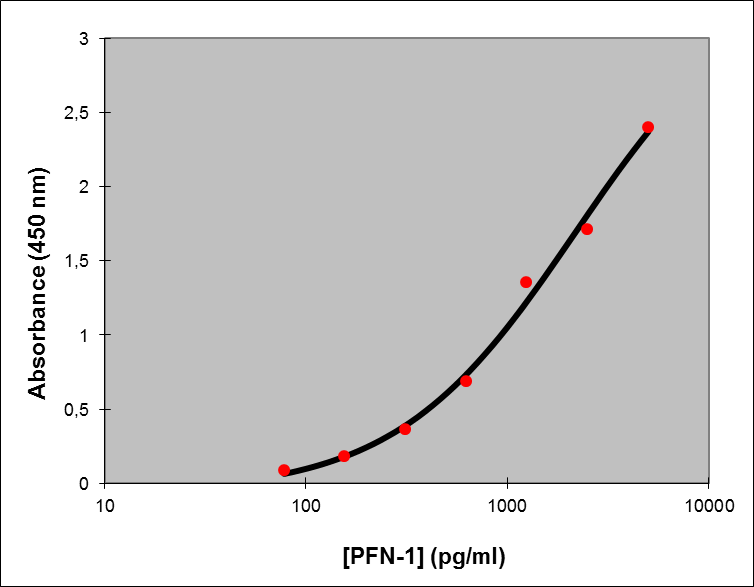
**

**Figure H. Standard curve validation of PFN-1.**

For NIF-1 (Cusabio Biotech CO. LTD, CSB-EL026683HU), a standard curve was obtained from 1 blank and 7 standards (25 to 1600pg/ml) that were analyzed in duplicates on 2 different days. The 4PL fit gave a R²=0.9966. (**Figure I**)

**
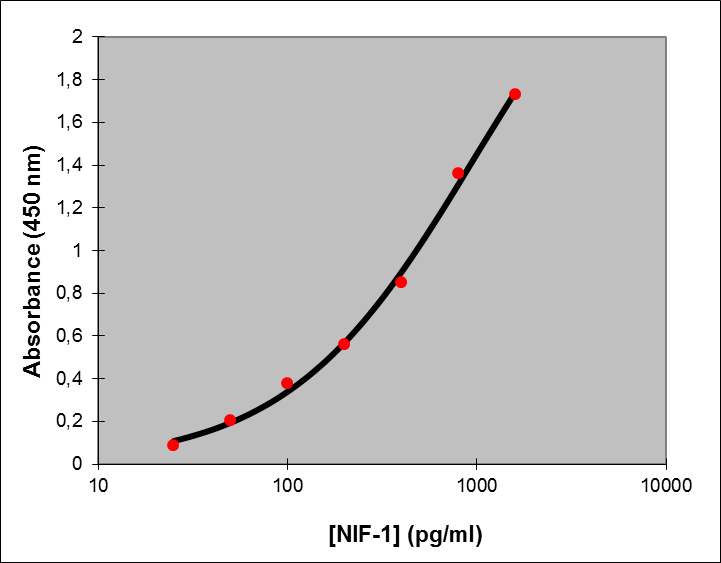
**

**Figure I** **Standard curve validation of NIF-1.**

For NIF-1 (USCN Life, WUHAN EIAAB SCIENCE CO. LTD, E1019h), standard curve was obtained from1 blank and 7 standards (0.31 to 20ng/ml) that were analyzed in duplicates on 2 different days. The 4PL fit gave a R²=0.999. (**Figure J**)

**
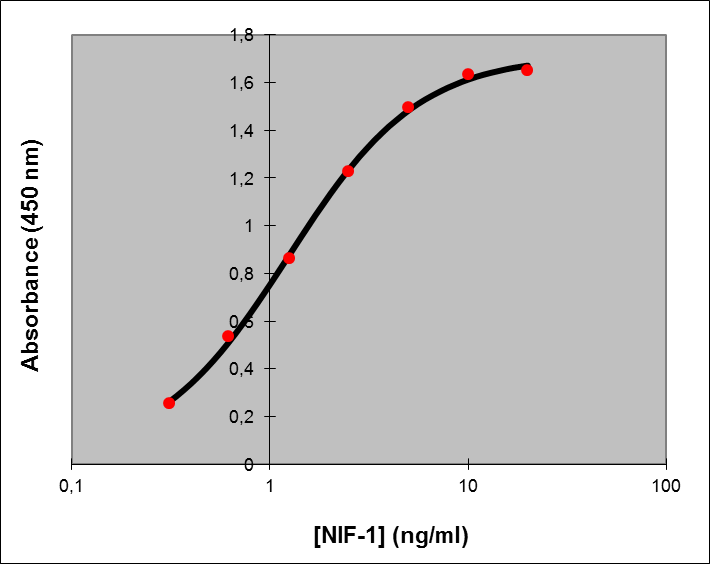
**

**Figure J.** **Standard curve validation of NIF-1.**
