# Supplementary material for: Analytical Performance of ELISA Assays in Urine: One More Bottleneck towards Biomarker Validation and Clinical Implementation
Source: PLoS One. 2016 Feb 18;11(2):e0149471. doi: 10.1371/journal.pone.0149471 (PMC4758723; doi:10.1371/journal.pone.0149471)
Supplement: S3 File — (DOCX) [file pone.0149471.s003.docx]

**Reproducibility**

For SPARC (R&D Systems Inc., DSP00), 3 samples with low, medium and high [SPARC] were assayed eight (8) times each. According to the 4PL fit, the CV of the low [SPARC] sample was 4%, of the medium 5% and of the high 8%. (**Fig. 2A**)

For PR3 (Cusabio Biotech CO. LTD, CSB-E13058h), 3 samples of low, medium and high [PR3] were assayed five (5) times each. (Fig.2B) According to the 4PL fit, the CV of the low [PR3] sample was 7%, of the medium [PR3] sample was 24% and of the high [PR3] sample was 21 %. (**Fig. 2B**)

For SLIT-2 (Cloud-Clone Corp. USCN Life Science Inc., SEA672Hu), 3 urine samples of low, medium and high [SLIT-2] were assayed eight (8) times each. The samples exhibited the following CVs: (18% for the medium [SLIT-2] sample, 18% for the high [SLIT-2] sample and 29% for the low [SLIT-2] sample. The medium and high [SLIT-2] samples had low CVs which were acceptable. The low [SLIT-2] sample had a high CV above the acceptable limit. According to the 4PL fit, the samples exhibited the following CVs: (19% for the medium [SLIT-2] sample, 44% for the high [SLIT-2] sample and 29% for the low [SLIT-2] sample. (**Figure A**)

**
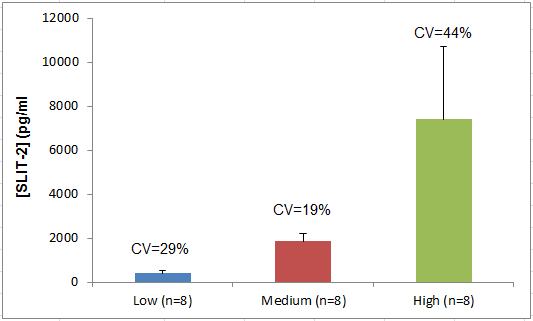
**

**Figure A. Reproducibility study results of SLIT-2.**

For H2B (US Biological Life Sciences, 025705), there was no high [H2B] sample available, therefore 2 samples of low [H2B] were assayed six (6) times each. The samples exhibited the following CVs: 42% and 33 % respectively for the 2 low [H2B] samples. Both samples had high CVs above the acceptable limit. According to the 4PL fit, CV could only be calculated for the one low [H2B] sample (56%). (**Figure B**)

**
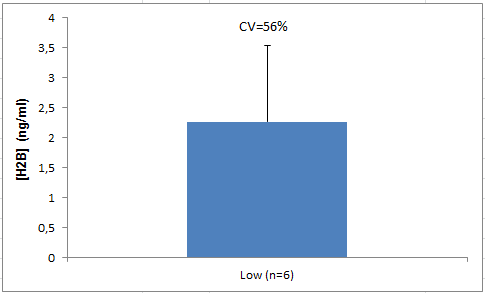
**

**Figure B.** **Reproducibility study results of H2B.**

For H2B (Cloud-Clone Corp. USCN Life Science Inc., SEA356Hu), there was no high [H2B] sample available, therefore 2 samples of low [H2B] were assayed six (6) times each. For the first low [H2B] sample, CV=97% and the second low [H2B] sample gave a negative result therefore CV could not be calculated. According to the 4PL fit, CV could be calculated only for the one low [H2B] sample (88%). (**Figure C**)

**
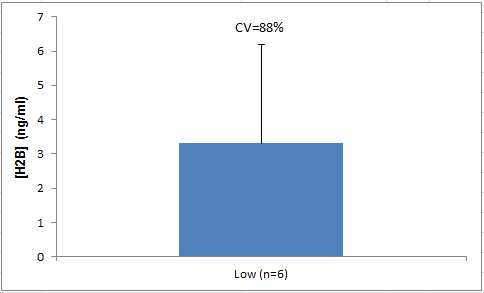
**

**Figure C.** **Reproducibility study results of H2B.**

For Survivin (Enzo Life Sciences, ADI-900-111), two samples of low and high [SURVIVIN]) were assayed twelve (12) times each. The samples exhibited the following CVs: 32% for the low [SURVIVIN] sample and 15% for the high [SURVIVIN] sample. The CV of the high [SURVIVIN] sample is acceptable whereas the CV of the low [SURVIVIN] sample was above the acceptable limit. According to the 4PL fit, CV could be calculated only for the high [SURVIVIN] sample (29%). (**Figure D**)

**
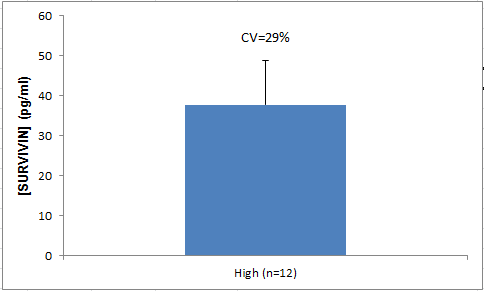
**

**Figure D.** **Reproducibility study results of SURVIVIN.**

For Survivin (R&D Systems Inc., DSV00), there was no high [SURVIVIN] sample available, therefore 2 samples of low [SURVIVIN] were assayed eight (8) times each. The first low [SURVIVIN] sample had CV=19% therefore satisfactory however the second gave a negative result. According to the 4PL fit, the first low [SURVIVIN] sample had CV=13% and the second low [SURVIVIN] sample had CV=6%.(**Figure E)**

**
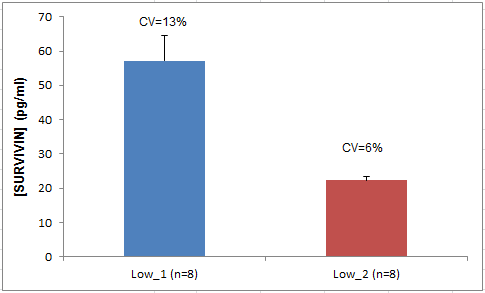
**

**Figure E.** **Reproducibility study results of SURVIVIN.**

For PFN-1 (USCN Life,WUHAN EIAAB SCIENCE CO. LTD, E2122h), 3 samples of low, medium and high [PFN-1] were assayed six (6) times each. The CV of the low concentration sample was not satisfactory (25%). The medium and high [PFN-1] samples exhibited % Recoveries of 17% and 15% respectively that were considered satisfactory.According to the 4PL fit, the CV of the low [PFN-1] sample was 39%, for the medium [PFN-1] sample was 16% and for the high [PFN-1] sample 55%.(**Figure F**)

**Figure F.** **Reproducibility study results of PFN-1.**

For PFN-1 (US Biological Life Sciences, 027613), 3 samples of low, medium and high [PFN-1] were assayed five (5) times each. The CV% was very satisfactory for the high [PFN-1] sample (3%) but out of the limits of acceptance for the medium and low [PFN-1] samples. (23% and 122% respectively)According to the 4PL fit, the 3 samples of low, medium and high [PFN-1] exhibited the same CVs. (**Figure G**)

**
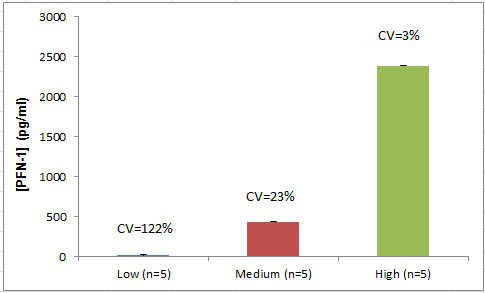
**

**Figure G.** **Reproducibility study results of PFN-1.**

For PFN-1 (Cloud-Clone Corp., USCN Life Science Inc., SEC233Hu), 3 samples of low, medium and high [PFN-1] were assayed six (6) times each. The low and high [PFN-1] samples exhibited satisfactory CVs (12% and 6% respectively), whereas the CV of the medium [PFN-1] sample was above the acceptable limit (23%).According to the 4PL fit, the low [PFN-1] sample had CV=6%, the medium and the high standards had CVs of 28% and 22% respectively). (**Figure H**)


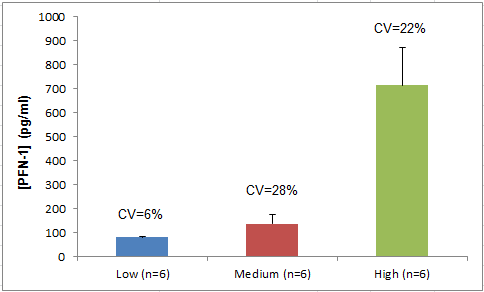


**Figure H.** **Reproducibility study results of PFN-1.**

For NIF-1 (CUSABIO Biotech CO. LTD, CSB-EL026683HU), 2 samples of medium and high [NIF-1] were assayed eight (8) times each. The samples exhibited the following CVs: (14% for the medium [NIF-1] sample and 6% for the high [NIF-1] sample). The high and the medium [NIF-1] samples had low CVs which were acceptable. According to the 4PL the medium and high [NIF-1] samples had CVs of 15% and 9% respectively. (**Figure I**)


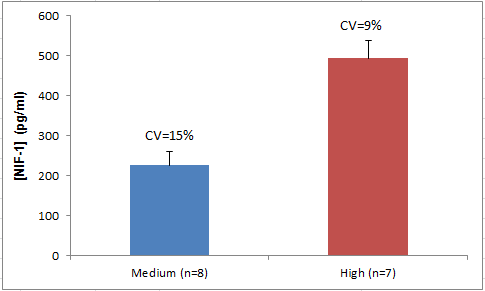


**Figure I.** **Reproducibility study results of NIF-1.**

For NIF-1 (USCN Life, WUHAN EIAAB SCIENCE CO., LTD, E1019h), 2 samples of low and medium [NIF-1] were assayed five (5) times each. Both samples exhibited satisfactory CVs. (10% for the low [NIF-1] sample and 11% for the medium [NIF-1] sample). According to the 4PL fit the low and medium [NIF-1] samples had CVs of 9% and 11% respectively. (**Figure J**)

**
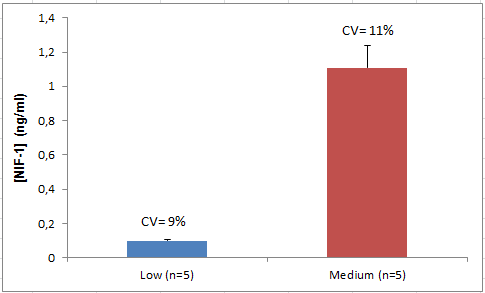
**

**Figure J.** **Reproducibility study results of NIF-1.**
