# Supplementary material for: Analytical Performance of ELISA Assays in Urine: One More Bottleneck towards Biomarker Validation and Clinical Implementation
Source: PLoS One. 2016 Feb 18;11(2):e0149471. doi: 10.1371/journal.pone.0149471 (PMC4758723; doi:10.1371/journal.pone.0149471)
Supplement: S4 File — (DOCX) [file pone.0149471.s004.docx]

**Linearity**

For SPARC (R&D Systems Inc., DSP00), a high [SPARC] sample ([SPARC] =31ng/ml) was diluted with sample diluent from 1:2 up to 1:16 and each dilution was assayed eight (8) times. (**Fig.3A**)

For PR3 (Cusabio Biotech CO., LTD, CSB-E13058h), a high [PR3] sample ([PR3] =8 ng/ml) was diluted with sample diluent from 1:2 up to 1:16 and each dilution was assayed five (5) times. (**Fig.3B**)

For SLIT-2 (Cloud-Clone Corp., USCN Life Science Inc., SEA672Hu), a high [SLIT-2] sample ([SLIT-2] =6189pg/ml) was diluted with sample diluent from 1:2 up to 1:16 and each dilution was assayed eight (8) times. R^2^=0.996 was satisfactory however the slope=0.557(<0.95) was not acceptable. (**Figure A**)

**Figure A. Linearity results of SLIT-2.**

For H2B (US Biological Life Sciences, 025705), there was no high [H2B] sample available therefore a urine sample with low [H2B], ([H2B] =4ng/ml) was diluted with sample diluent to 1:4 and the dilution was assayed six (6) times. The minimum detectable dose (MDD) was 1.28 ng/ml and the 1:2 and 1:4 dilutions of the low [H2B] sample yielded a negative result thus a linearity test could not be performed on clinical urine samples.

For H2B (Cloud-Clone Corp., USCN Life Science Inc., SEA356Hu), there was no high [H2B] sample available therefore a urine sample with low [H2B], ([H2B] =2.25ng/ml) was diluted with sample diluent to 1:4 and the dilution was assayed six (6) times. The minimum detectable dose (MDD) was 1.33 ng/ml the 1:4 dilution of the low [H2B] sample yielded a negative result thus a linearity test could not be performed on clinical urine samples.

For Survivin (Enzo Life Sciences, ADI-900-111), a high [SURVIVIN] sample ([SURVIVIN] =82pg/ml) was diluted with sample diluent from 1:2 up to 1:16 and each dilution was assayed five (5) times. The R^2^=0.993 was satisfactory but the slope=3.1469 was not acceptable. Therefore the linearity test for SURVIVIN did not yield satisfactory results. (**Figure B)**

**Figure B.** **Linearity results of SURVIVIN.**

For Survivin (R&D Systems Inc., DSV00), since there was not a sample with high [SURVIVIN] available, a urine sample with low [SURVIVIN] ([SURVIVIN] =51pg/ml) was diluted with sample diluent from 1:2 to 1:4 and each dilution was assayed eight (8) times. The minimum detectable dose (MDD) was 9.96pg/ml and the 1:2 to 1:4 dilutions of the low [SURVIVIN] sample yielded a negative result therefore a linearity test could not be performed on clinical urine samples.

For PFN-1 (USCN Life, WUHAN EIAAB SCIENCE CO. LTD, E2122h), a high [PFN-1] sample ([PFN-1] =3973 pg/ml) was diluted with sample diluent from 1:2 up to 1:32 and each dilution was assayed five (5) times. The R^2^=0.9943 was satisfactory however the slope=1.2847 (>1.05) was not acceptable. Therefore the linearity test for PFN-1 did not yield satisfactory results. (**Figure C)**

**Figure C.** **Linearity results of PFN-1.**

For PFN-1 (US Biological Life Sciences, 027613), a high [PFN-1] sample ([PFN-1] =2233 pg/ml) was diluted with sample diluent from 1:2 up to 1:32 and each dilution was assayed five (5) times. The R^2^=0.362 and the slope=0.8305 (<0.95) were not satisfactory. Therefore the linearity test for PFN-1 did not yield satisfactory results. (**Figure D)**

**Figure D.** **Linearity results of PFN-1.**

For PFN-1 (Cloud-Clone Corp., USCN Life Science Inc., SEC233Hu), a high [PFN-1] sample ([PFN-1]=1088 pg/ml) was diluted with sample diluent from 1:2 up to 1:8 and each dilution was assayed four (4) times. The R^2^=0.259 and the slope=-1.5603 (<0.95) were not satisfactory, therefore the linearity test did not yield satisfactory results. (**Figure E)**

**Figure E.** **Linearity results of PFN-1.**

For NIF-1 (CUSABIO, CSB-EL026683HU), a high [NIF-1] sample ([NIF-1] =613 pg/ml) was diluted with sample diluent from 1:2 up to 1:32 and each dilution was assayed five (5) times. The R^2^=0.924 and the slope=1.5986 (>1.05) were not satisfactory, therefore the linearity test for NIF-1 did not yield satisfactory results. (**Figure F)**

**Figure F. Linearity results of NIF-1.**

For NIF-1 (USCN Life, WUHAN EIAAB SCIENCE CO., LTD, E1019h), a medium [NIF-1] sample ([NIF-1] =3 ng/ml) was diluted with sample diluent from 1:2 up to 1:16 and each dilution was assayed five (5) times. The R^2^=0.696 and the slope=1.1752 (>1.05) were not satisfactory, therefore the linearity test for NIF-1 did not yield satisfactory results. (**Figure G)**

**Figure G.** **Linearity results of NIF-1.**
