# Supplementary material for: Analytical Performance of ELISA Assays in Urine: One More Bottleneck towards Biomarker Validation and Clinical Implementation
Source: PLoS One. 2016 Feb 18;11(2):e0149471. doi: 10.1371/journal.pone.0149471 (PMC4758723; doi:10.1371/journal.pone.0149471)
Supplement: S5 File — (DOCX) [file pone.0149471.s005.docx]

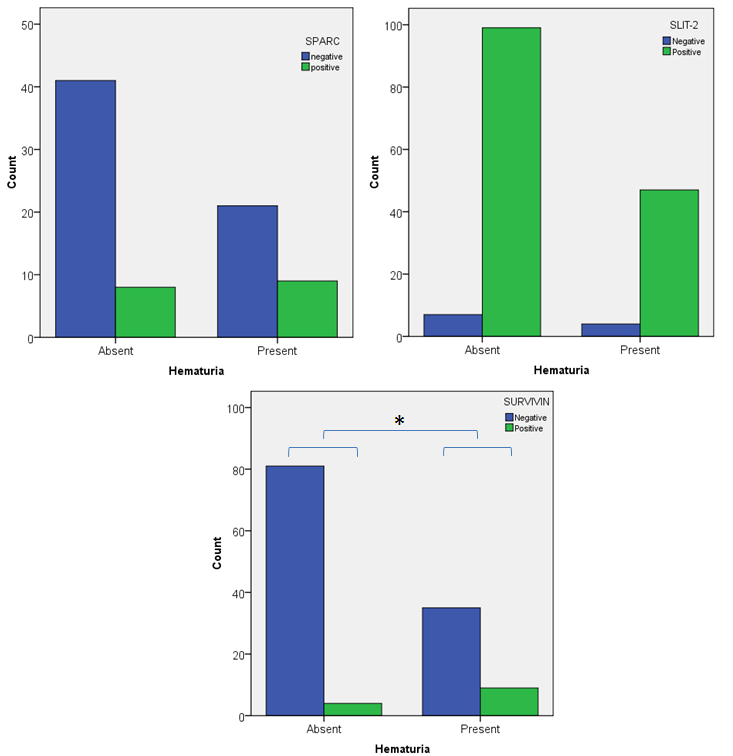


**Figure A. Bar plot results of SPARC (R&D Systems), SLIT-2 (Cloud Clone Corp.) and SURVIVIN (R&D Systems) ELISA (positive/negative) relative to the presence/absence of hematuria. (*p<0.05)**
