# Supplementary material for: Analytical Performance of ELISA Assays in Urine: One More Bottleneck towards Biomarker Validation and Clinical Implementation
Source: PLoS One. 2016 Feb 18;11(2):e0149471. doi: 10.1371/journal.pone.0149471 (PMC4758723; doi:10.1371/journal.pone.0149471)
Supplement: S6 File — (DOCX) [file pone.0149471.s006.docx]

**Figure A. ELISA results of SPARC relative to cancer grade.**

**Figure B. ELISA results of SLIT-2 relative to cancer grade.**
