# Supplementary material for: Analytical Performance of ELISA Assays in Urine: One More Bottleneck towards Biomarker Validation and Clinical Implementation
Source: PLoS One. 2016 Feb 18;11(2):e0149471. doi: 10.1371/journal.pone.0149471 (PMC4758723; doi:10.1371/journal.pone.0149471)
Supplement: S7 File — (DOCX) [file pone.0149471.s007.docx]

**Table A. Urine strip analysis, ELISA results, and clinical data for urine samples**

| Sample | Sex | Age (years) | Hematuria qualitative result: negative (ng), +, ++, +++) | pH | SPARC (ng/ml) | SLIT-2 (pg/ml) | SURVIVIN (pg/ml) | Clinical information |
| --- | --- | --- | --- | --- | --- | --- | --- | --- |
| L_B11 | M | 70 | ng | 6 |  | 2887 | 8.23 | Benign Prostate Hyperplasia (BPH) |
| L_B17 | M | 49 | ng | 6 | 0 | 2181 | 0 | HERNIA |
| L_B18 | M | 68 | ng | 6.5 |  | 0 | 0 | BPH |
| L_B19 | M | 69 | ng | 6 | 2.64 | 1316 |  | PYELONEPHRITIS |
| L_B21 | F | 73 | ng | 6 |  | 78 | 0 | CYSTITIS |
| L_B22 | M | 77 | Hematuria (+) | 6 | 2.01 | 10082 |  | BPH |
| L_B26 | M | 81 | ng | 8 |  | 637 | 0 | BPH |
| L_B29 | F | 62 | ng | 6 |  | 4931 | 0 | HEMATURIA |
| L_B30 | M | 22 | ng | 6 | 0 | 4391 | 0 |  |
| LB31 | M | 75 |  |  | 3.97 | 1968 |  | BPH |
| L_B32 | M | 42 | ng | 6 |  | 1070 |  | MARION DISEASE |
| L_B34 | F | 44 | ng | 7 | 0 | 441 | 0 | HEMATURIA |
| L_B35 | M | 74 | ng | 6 |  | 2191 | 0 | HEMATURIA |
| L_B37 | M | 71 | ng | 6 | 0 | 2252 | 0 | BPH |
| L_B38 | F | 46 | ng | 6 |  | 2128 | 0 | CYSTITIS |
| L_B42 | M | 73 | ng | 6.5 |  | 85 |  | BPH |
| L_B43 | M | 88 | ng | 6 |  | 2249 |  | RETROPERITONEAL FIBROSIS |
| L_B44 | M | 72 | ng | 6 |  | 792 | 0 | URETERAL LITHIASIS |
| L_B47 | M | 68 | ng | 6.5 |  | 694 | 0 | Inflammation |
| L_B49 | M | 67 | ng | 6.5 | 0 | 1534 | 0 | BPH |
| L_B53 | M | 66 | Hematuria (+++) | 7 |  | 0 | 5.31 | microscopic hematuria |
| L_B54 | M | 78 | ng | 6 | 0 | 1381 | 0 | LITHIASIS URETER |
| L_B55 | M | 84 | Hematuria (+) | 6 | 0 | 2202 | 0 | BPH |
| L_B57 | M | 29 | ng | 6 |  | 486 | 0 | varicocele |
| L_B59 | M | 72 | ng | 6 |  | 2750 |  | BPH |
| L_B63 | M | 70 | ng | 6.5 | 0 | 399 | 11.4 | BPH |
| L_B71 | M | 69 | ng | 6 | 0 | 2523 | 0 | BPH |
| L_B85 | M | 68 | ng | 6 |  | 587 |  | HEMATURIA |
| L_B88 | M | 59 | ng | 6.5 |  | 379 | 0 | BPH |
| L_B107 | M | 64 | Hematuria (+++) | 6 |  | 559 |  | BPH and chronic inflammation |
| L_B109 | M | 53 | Hematuria (++) | 8 |  |  |  | Inflamation |
| L_B111 | M | 52 | ng | 6 | 0 | 113 | 0 | PEYRONIE DISEASE |
| L_B114 | M | 84 | ng | 6.5 |  | 423 | 0 | BPH |
| L_B119 | M | 58 | ng | 6 |  | 294 |  | hydrocele |
| L_B124 | F | 77 | Hematuria (+++) | 6 |  | 320 | 0 |  |
| L_B128 | F | 63 |  |  |  | 0 |  |  |
| L_B131 | M | 75 | ng | 6 |  | 878 | 0 | papilloma |
| L_B140 | M | 76 | Hematuria (+) | 6 | 0 | 1311 | 0 | BPH and prostatitis |
| L_B142 | M | 75 | ng | 6 |  | 837 | 0 | HERNIA |
| L_B143 | M | 59 | ng | 6 |  | 0 |  | hyperplasia, chronic prostatitis |
| L_B144 | M | 74 | ng | 6 |  | 490 |  | BPH |
| L_B145 | M | 68 | ng | 6 |  | 275 |  |  |
| L_B148 | M | 72 | ng | 6 |  | 2911 | 0 | varicocele |
| L_B149 | M | 29 | ng | 6 | 0 | 188 | 0 | LITHIASIS |
| L_B151 | M | 59 | ng | 6 | 0 | 4388 | 0 | BPH |
| L_B152 | M | 65 | ng | 6.5 | 0 | 267 | 0 | nodular hyperplasia, prostatitis |
| L_B153 | M | 73 | ng | 6 | 0 | 1240 | 0 | BPH |
| L_B158 | M | 68 | ng | 6.5 | 2.01 | 1044 | 0 | inflammation |
| L_B163 | M | 81 | ng | 6 | 0 | 109 | 0 | BPH |
| L_B164 | F | 69 | ng | 6 |  | 517 |  |  |
| L_B171 | M | 60 | ng | 6 |  | 354 | 0 | polypoid cystitis |
| A_B10 | M | 81 | Hematuria (+++) | 7 |  | 2501 |  | HEMATURIA |
| A_B20 | M | 83 | ng | 6 |  | 285 |  |  |
| L_Ta4 | M | 69 | Hematuria (++) | 6.5 |  | 728 | 0 | TaG2 |
| L_Ta5 | F | 39 | ng | 6 | 0 | 2435 | 0 | pTa |
| L_Ta8 | M | 79 | ng | 6 | 0 | 5167 | 0 | TaG2 |
| L_Ta10 | M | 70 | ng | 7 | 0 | 1383 | 0 | TaG1 |
| L_Ta13 | M | 60 | ng | 6 | 0 | 2961 | 0 | TaG2 |
| L_Ta16 | M | 72 | ng | 6 | 0 | 3204 | 0 | TaG2 |
| L_Ta25 | F | 74 | ng | 6.5 | 0 | 68 | 0 | TaG2 |
| L_Ta45 | M | 80 | ng | 6 | 0 | 2042 | 0 | TaG1 |
| L_Ta48 | M | 79 | Non Hematuria (+) | 6 | 3.72 | 2307 | 0 | TaG2 |
| L_Ta51 | M | 76 | Non Hematuria (++) | 7 | 0 | 0 | 0 | TaG2 |
| L_Ta52 | M | 60 | Non Hematuria (+) | 7 | 0 | 1060 | 0 | TaG1 |
| L_Ta56 | M | 74 | Hematuria (+) | 6 | 0 | 24 | 0 | TaG3 |
| L_Ta61 | M | 72 | ng | 6 | 0 | 1846 |  | TaG2 |
| L_Ta72 | F | 57 | ng | 6 |  | 6206 | 0 | TaG1 |
| L_Ta78 | M | 77 | Hematuria (+) | 6 | 0 | 3398 | 0 | TaG2 |
| L_Ta83 | M | 83 | ng | 6 | 0 | 617 | 0 | TaG2 |
| L_Ta104 | M | 71 | ng | 9 | 0 | 0 | 0 | TaG1 |
| L_Ta112 | M | 63 | ng | 6.5 |  | 857 | 0 | TaG1-2 |
| L_Ta113 | M | 76 | Hematuria (+) | 6 |  | 1076 | 0 | TaG1 |
| L_Ta115 | F | 66 | ng | 6.5 |  | 1529 | 0 | TaG1 |
| L_Ta116 | M | 52 | ng | 6 |  | 2409 | 0 | TaG3 |
| L_Ta117 | M | 68 | Hematuria (+) | 6 |  | 1142 | 0 | TaG1 |
| L_Ta118 | M | 60 | ng | 6.5 | 0 | 435 | 4.54 | TaG2-3 |
| L_Ta121 | F | 76 | Non Hematuria (+) | 6 |  | 1047 |  | TaG1 |
| L_Ta123 | F | 67 | ng | 6 |  | 894 | 0 | pTa |
| L_Ta126 | M | 82 | ng | 6 | 0 | 1274 | 0 | TaG1 |
| L_Ta129 | M | 53 | ng | 7 |  | 0 | 0 | TaG1 |
| L_Ta134 | M | 59 | Hematuria (+++) | 6.5 |  | 2170 | 71.3 | TaG1 |
| L_Ta146 | M | 51 | ng | 6 |  | 2454 | 0 | TaG1 |
| L_Ta147 | M | 64 | Hematuria (+) | 6 |  | 3311 | 0 | TaG1 |
| L_Ta154 | F | 79 |  |  |  | 75 |  | TaG1 |
| L_Ta155 | M | 68 | ng | 6 |  | 322 | 0 | TaG1 |
| L_Ta157 | M | 63 | Hematuria (+++) | 6.5 | 1.26 | 956 | 0 | TaG1 |
| L_Ta160 | M | 77 | Hematuria (++) | 6.5 | 0 | 702 | 0 | TaG2 |
| L_Ta161 | M | 60 | ng | 6 |  | 800 | 0 | TaG1 |
| L_Ta162 | M | 75 | ng | 6 | 0 | 636 | 0 | TaG1 |
| L_Ta166 | M | 66 | ng | 7 | 0 | 988 | 0 | TaG2 |
| L_Ta168 | F | 64 | ng | 6 |  | 165 | 0 | TaG2 |
| A_Ta1 | M | 68 | ng | 6 |  | 0 |  | TaG2 |
| A_Ta15 | M | 62 | Non Hematuria (+) | 6 |  | 7247 |  | TaG2 |
| A_Ta16 | M | 61 | Hematuria (++) | 6 |  | 222 |  | TaG2 |
| A_Ta17 | M | 88 | ng | 6 |  | 65 |  | TaG2 |
| A_Ta22 | M | 85 | ng | 7 |  | 438 |  | TaG2 |
| A_Ta23 | F | 84 | Non Hematuria (+) | 6 |  | 614 |  | TaG2 |
| A_Ta24 | M | 84 | Hematuria (+++) | 7 |  | 3299 |  | TaG2 |
| A_Ta32 | M | 66 | ng | 6 |  | 0 |  | TaG2 |
| L_T(1) 2 | F | 54 | ng | 6 |  | 3718 | 0 | T1 |
| L_T(1) 3 | M | 70 | ng | 6 |  | 3318 | 0 | T1G2 |
| L_T(1) 6 | F | 81 | ng | 6 |  | 3994 | 0 | T1G2 |
| L_T(1) 7 | M | 86 | Hematuria (+) | 6 |  | 7944 | 0 | T2G2 |
| L_T(1) 33 | M | 58 | ng | 6 |  | 2596 | 0 | T1G3 |
| L_T(1) 41 | M | 70 | ng | 8 |  | 1942 | 0 | T1G2 |
| L_T(1) 50 | M | 66 | ng | 6.5 |  | 318 | 0 | T1G3 |
| L_T(1) 62 | M | 74 | Hematuria (+++) | 6 | 0 | 1881 | 0 | T1G3 |
| L_T(1) 65 | F | 63 | ng | 6 |  | 1075 | 0 | T1G2 |
| L_T(1) 66 | M | 72 | Hematuria (++) | 6 | 0 | 2794 | 0 | T1G3 |
| L_T(1) 67 | M | 67 | ng | 6 |  | 1088 | 0 | T1G3 |
| L_T(1) 73 | F | 57 | Hematuria (+++) | 6.5 |  | 1614 | 0 | T1G3 |
| L_T(1)74 | M | 83 | ng | 7 | 0 | 139 | 0 | T1G3 |
| L_T(1) 84 | M | 67 | ng | 6 | 0 | 5174 | 0 | T1G3 |
| L_T(1) 95 | M | 67 | ng | 6 | 10.6 | 948 | 0 | T1G2 |
| L_T(1) 98 | F | 54 | ng | 6 | 0 | 2098 | 0 | T1G2 |
| L_T(1) 99 | M | 60 | Hematuria (+++) | 6 |  | 5017 | 0 | T1G3 |
| L_T(1)125 | M | 69 | ng | 6 | 0 | 1102 | 0 | T1G2-3 |
| L_T(1) 133 | F | 52 | Hematuria (+) | 6 | 0 | 768 | 0 | T1G2-3 |
| L_T(1) 138 | M | 73 | Hematuria (+) | 6 | 0 | 2611 | 0 | T1G2-3 |
| L_T(1) 172 | M | 49 |  |  | 0 | 3711 | 0 | T1G3 |
| L_T(1) 173 | M | 71 | ng | 8 | 0 | 0 | 0 | Τ1G2-3 |
| L_T(1) 174 | M | 55 | Hematuria (+) | 6 | 0 | 3084 | 0 | T1G2-3 |
| A_T(1) 3 | M | 72 | ng | 6.5 | 0.79 | 659 | 0 | T1G2 |
| A_T(1) 5 | M | 60 | ng | 6.5 | 1.26 | 97 | 0 | T1G3 |
| A_T(1) 6 | M | 82 | ng | 6 | 0 | 562 | 0 | T1G2 |
| A_T(1) 7 | M | 80 | Hematuria (+) | 6 | 0 | 1153 | 0 | T1G2 |
| A_T(1) 9 | M | 68 | ng | 6 | 0 | 819 |  | T1G3 |
| A_T(1)11 | M | 90 | Hematuria (+) | 8 |  | 509 | 0 | T1G2-3 |
| A_T(1) 12 | M | 86 | Hematuria (+) | 8 | 2.64 | 0 | 0 | T1G2 |
| A_T(1) 13 | M | 85 | ng | 6 | 0 | 1867 | 0 | T1G2-3 |
| A_T(1) 14 | M | 73 | Hematuria (+++) | 6.5 |  | 107 | 0 | T1G2-3 |
| A_T(1) 21 | M | 76 | Hematuria (+++) | 6.5 |  | 399 | 0 | T1G2-3 |
| A_T(1) 25 | M | 63 | Non Hematuria (+) | 6 | 0 | 160 | 0 | T1G3 |
| A_T(1) 27 | M | 82 | ng | 6 |  | 8349 | 0 | T1G3 |
| A_T(1) 30 | M | 80 | ng | 7 |  | 364 | 0 | T1G3 |
| L_T(4)1 | M | 70 | ng | 8 | 5.74 | 1759 | 0 | T4 |
| L_T(2)9 | M | 86 | Hematuria (+) | 6.5 | 0 | 0 | 0 | T2G3 |
| L_T(2)14 | M | 80 | ng | 6 |  | 1609 |  | T2 |
| L_T(2)15 | M | 70 | Non Hematuria (+) | 6 |  |  |  | T2G3 |
| L_T(2)20 | M | 69 | Hematuria (+++) | 6 | 0 | 7311 | 14.6 | T2G3 |
| L_T(2)27 | M | 77 | ng | 6 |  | 570 | 2.65 | T2G3 |
| L_T(2)36 | M | 74 | ng | 6 | 0 | 2357 | 0 | T2 |
| L_T(2)69 | M | 78 |  |  |  | 8984 |  | T2G3 |
| L_T(3)70 | M | 73 | Non Hematuria (+) | 6 | 0 | 437 | 0 | T3 |
| L_T(2)76 | M | 69 | ng | 6 |  | 2496 | 0 | T2 |
| L_T(2)77 | M | 69 | Hematuria (+++) | 7 |  |  | 410 | T2 |
| L_T(23)80 | M | 76 | ng | 6 | 1.26 | 2785 | 0 | T3 |
| L_(T2)90 | M | 49 | Hematuria (++) | 6.5 |  | 1377 | 2.65 | T2 |
| L_T(23)93 | M | 74 | ng | 6 |  | 2507 | 0 | T2-3 |
| L_T(4)94 | M | 74 | Hematuria (+) | 6 | 0 | 2301 | 0 | T4G3 |
| L_T(4)100 | M | 65 | Hematuria (+++) | 6 | 1.65 | 7568 | 10.4 | T4 |
| L_T(23)101 | M | 68 | ng | 8 |  | 211 | 0 | T2-3 |
| L_(T3)102 | F | 71 | Hematuria (+++) | 6.5 | 7.80 | 39848 | 27.1 | T3 |
| L_T(3 )106 | M | 70 | ng | 6 | 0 | 6324 | 0 | T3G3 |
| L_T(2) 108 | M | 56 | Hematuria (+) | 7 |  | 19113 | 30.9 | T2 |
| L_T(4) 110 | M | 79 | Hematuria (+) | 6 | 0 | 189 | 0 | T4 |
| L_T(4) 122 | F | 70 | ng | 6 |  | 3246 | 0 | T4 |
| L_T(2) 130 | M | 75 | ng | 6 | 0 | 4213 | 0 | T2 |
| L_T(2) 132 | M | 27 | Hematuria (+++) | 6 | 19.10 | 16512 | 82.6 | T2 |
| L_T(2)136 | M | 61 | Hematuria (+) | 6 | 1.65 | 3633 | 0 | T2 |
| L_T(2) 150 | F | 65 | Hematuria (++) | 6 | 16.60 |  | 0 | T2 |
| L_T(2) 165 | M | 54 | Hematuria (++) | 6 | 0 | 1108 | 0 | T2 |
| L_T(2)167 | M | 66 |  |  | 7.45 | 4534 | 0 | T2G3 |
| A_T(3)4 | M | 73 | ng | 6 | 0 | 198 |  | T3G3 |
| A_T(4)18 | M | 78 | Hematuria (+++) | 6 | 0 | 1568 |  | T4G2-3 |
| A_T(3)19 | M | 53 | ng | 6 | 0 | 1289 |  | T3G3 |
| A_T(2)26 | M | 72 | ng | 6.5 | 20.0 | 433 | 0 | T2G3 |
