# Supplementary material for: Analytical Performance of ELISA Assays in Urine: One More Bottleneck towards Biomarker Validation and Clinical Implementation
Source: PLoS One. 2016 Feb 18;11(2):e0149471. doi: 10.1371/journal.pone.0149471 (PMC4758723; doi:10.1371/journal.pone.0149471)
Supplement: S8 File — (DOCX) [file pone.0149471.s008.docx]

**Recovery**

For SPARC (R&D Systems Inc., DSP00),spike experiments were performed by adding 50μl of a urine sample, negative for SPARC, to 50μl of three different standards of low, medium and high [SPARC] in eight (8) replicates. (Low =3.13 ng/ml, medium=12.5 ng/ml and high=50 ng/ml). (**Table. 2**)

For PR3 (Cusabio Biotech CO. LTD, CSB-E13058h), spike experiments were performed by adding 50μl of a urine sample, negative for PR3 to 50μl of three different standards of low, medium and high [PR3] in four (4) replicates. (Low=1.56 ng/ml, medium=6.25 ng/ml and high=25 ng/ml. (**Table. 3**)

For SLIT-2 (Cloud-Clone Corp., USCN Life Science Inc., SEA672Hu), no negative sample could be made readily available so the spiked experiments were prepared by adding 50μl of normal urine ([SLIT-2] =1205pg/ml) to 50μl of three different standards of low, medium and high [SLIT-2] (low=78 pg/ml, medium=625 pg/ml and high=5000pg/ml). The spiked samples were analyzed six (6) times each. Τhe % Recovery of the low standard was 82%, for the medium 71% and for the high 99%. Thus spiking [SLIT-2] standards in urine yielded satisfactory results. According to the 4PL fit, the % Recovery of the low standard was 57%, for the medium 50% and for the high 69%. **(Table A)**

**Table A. Recovery study results of SLIT-2**

| Normal (1205 pg/ml) + 78 pg/ml SLIT-2 (n=6) | Mean [SLIT-2] (pg/ml) | 369 |
| --- | --- | --- |
|  | Expected [SLIT-2] (pg/ml) | 642 |
|  | % Recovery | ***57%*** |
| Normal (1205 pg/ml) + 625 pg/ml SLIT-2 (n=6) | Mean [SLIT-2] (pg/ml) | 457 |
|  | Expected [SLIT-2] (pg/ml) | 915 |
|  | % Recovery | ***50%*** |
| Normal (1205 pg/ml) + 5000 pg/ml SLIT-2 (n=6) | Mean [SLIT-2] (pg/ml) | 2150 |
|  | Expected [SLIT-2] (pg/ml) | 3103 |
|  | % Recovery | **69%** |

For H2B (US Biological Life Sciences, 025705), spike experiments were performed by adding 50μl of a urine sample negative for H2B to 50μl of three different standards of low, medium and high [H2B] in six (6) replicates. (Low= 6.25 pg/ml, medium=25 ng/ml, and high=200 ng/ml)The % Recovery for the high standard was 298% and for the low and the medium standard 64% and 165% respectively. Thus spiking [H2B] standards in urine did not yield satisfactory results. According to the 4PL fit, the % recovery could only be calculated for the medium standard (120%). (**Table B**)

**Table B. Recovery study results of H2B**

| Negative+ 6.25 ng/ml H2B (n=6) | Mean [H2B] (ng/ml) | N/A |
| --- | --- | --- |
|  | Expected [H2B] (ng/ml) | 3.125 |
|  | % Recovery | ***N/A*** |
| Negative + 25 ng/ml H2B (n=6) | Mean [H2B] (ng/ml) | 15 |
|  | Expected [H2B] (ng/ml) | 12.5 |
|  | % Recovery | ***120%*** |
| Negative + 200 ng/ml H2B (n=6) | Mean [H2B] (ng/ml) | N/A |
|  | Expected [H2B] (ng/ml) | 100 |
|  | % Recovery | ***N/A*** |

For H2B (Cloud-Clone Corp., USCN Life Science Inc., SEA356Hu), spike experiments were performed by adding 50μl of a urine sample, negative for [H2B] to 50μl of three different standards of low, medium and high [H2B] in duplicates. (Low=3.12 ng/ml, medium= 12.5 ng/ml and high= 100ng/ml) The % Recovery of the low standard was 0%, for the medium 43% and for the high 150%. Thus spiking [H2B] standards in urine did not yield a satisfactory result. According to the 4PL fit, the % recoveries could be calculated only for the medium and high standards (11% and 74% respectively). (**Table C**)

**Table C.** **Recovery study results of H2B**

| Negative + 3.12 ng/ml H2B (n=2) | Mean [H2B] (ng/ml) | N/A |
| --- | --- | --- |
|  | Expected [H2B] (ng/ml) | 1.56 |
|  | % Recovery | ***N/A*** |
| Negative + 12.5 ng/ml H2B (n=2) | Mean [H2B] (ng/ml) | 0.68 |
|  | Expected [H2B] (ng/ml) | 6.25 |
|  | % Recovery | ***11%*** |
| Negative + 100 ng/ml H2B (n=2) | Mean [H2B] (ng/ml) | 37 |
|  | Expected [H2B] (ng/ml) | 50 |
|  | % Recovery | ***74%*** |

For Survivin (Enzo Life Sciences, ADI-900-111) spike experiments were performed by adding 50μl of a urine sample negative for SURVIVIN to 50μl of three different standards of low, medium and high [SURVIVIN] in triplicates. (Low=62.5 pg/ml, medium= 250 pg/ml and high=1000pg/ml) The % Recovery for the low standard was 256%, for the medium 207% and for the high 198%. All standards exhibited recoveries higher than the manufacturer claimed for urine (88.1%). Thus spiking [SURVIVIN] standards in urine did not yield satisfactory results. According to the 4PL fit, the % recovery of the low [SURVIVIN] standard was 106%, of the medium [SURVIVIN] standard was 124% and of the high [SURVIVIN] standard was 114%. (**Table D**)

**Table D. Recovery study results of SURVIVIN**

| Negative + 62.5 pg/ml SURVIVIN (n=6) | Mean [SURVIVIN] (pg/ml) | 33 |
| --- | --- | --- |
|  | Expected [SURVIVIN] (pg/ml) | 31.25 |
|  | % Recovery | ***106%*** |
| Negative + 250 pg/ml SURVIVIN (n=6) | Mean [SURVIVIN] (pg/ml) | 155 |
|  | Expected [SURVIVIN] (pg/ml) | 125 |
|  | % Recovery | ***124%*** |
| Negative + 1000 pg/ml SURVIVIN (n=6) | Mean [SURVIVIN] (pg/ml) | 568 |
|  | Expected [SURVIVIN] (pg/ml) | 500 |
|  | % Recovery | ***114%*** |

For Survivin (R&D Systems Inc., DSV00),spike experiments were performed by adding 50μl of a urine sample negative for SURVIVIN to 50μl of three different standards of low, medium and high [SURVIVIN] in five (5) replicates (Low=62.5 pg/ml, medium= 250 pg/ml and high=1000pg/ml. The % Recovery for the low standard was 82%, for the medium standard 70% and for the high standard 91%. Thus spiking [SURVIVIN] standards in urine yielded satisfactory results. According to the 4PL fit, the % Recovery for the low standard was 122%, for the medium standard 68% and for the high standard 82%. (**Table E**)

**Table E. Recovery study results of SURVIVIN**

| Negative + 62.5 pg/ml SURVIVIN (n=5) | Mean [SURVIVIN] (pg/ml) | 38 |
| --- | --- | --- |
|  | Expected [SURVIVIN] (pg/ml) | 31.25 |
|  | % Recovery | ***122%*** |
| Negative + 250 pg/ml SURVIVIN (n=5) | Mean [SURVIVIN] (pg/ml) | 85 |
|  | Expected [SURVIVIN] (pg/ml) | 125 |
|  | % Recovery | ***68%*** |
| Negative + 1000 pg/ml SURVIVIN (n=5) | Mean [SURVIVIN] (pg/ml) | 410 |
|  | Expected [SURVIVIN] (pg/ml) | 500 |
|  | % Recovery | ***82%*** |

For PFN-1 (USCN Life,WUHAN EIAAB SCIENCE CO. LTD, E2122h), spike experiments were performed by adding 50μl of a urine sample, negative for PFN-1 to 50μl of three different standards of low, medium and high [PFN-1] in four (4) replicates. (Low=156 pg/ml, medium=625 pg/ml and high=5000 pg/ml). According to the 4PL fit, the % recovery for the low standard was 54%, for the medium 24% and for the high 13%. Thus spiking [PFN-1] standards in urine did not yield satisfactory results. (**Table F**)

**Table F. Recovery study results of PFN-1**

| Negative + 5000 pg/ml PFN-1 (n=4) | Mean [PFN-1] (pg/ml) | 326 |
| --- | --- | --- |
|  | Expected [PFN-1] (pg/ml) | 2500 |
|  | % Recovery | ***13%*** |
| Negative + 625 pg/ml PFN-1 (n=4) | Mean [PFN-1] (pg/ml) | 74 |
|  | Expected [PFN-1] (pg/ml) | 312.5 |
|  | % Recovery | ***24%*** |
| Negative + 156 pg/ml PFN-1 (n=4) | Mean [PFN-1] (pg/ml) | 42 |
|  | Expected [PFN-1] (pg/ml) | 78 |
|  | % Recovery | ***54%*** |

For PFN-1 (US Biological Life Sciences, 027613), spike experiments were performed by adding 50μl of a urine sample, negative for PFN-1 to 50μl of three different standards of low, medium and high [PFN-1] in five (5) replicates. (Low=156 pg/ml, medium=625 pg/ml and high=2500 pg/ml) The % Recovery was very low and not acceptable for the low standard (13%) while it was quite satisfactory for the medium and high standards. (95% and 112% respectively) Thus spiking [PFN-1] standards in urine did not yield satisfactory results. According to the 4PL fit, % Recovery could only be calculated for the medium and high standards (94% and 113% respectively). (**Table G**)

**Table G. Recovery study results of PFN-1**

| Negative + 156 pg/ml PFN-1 (n=5) | Mean [PFN-1] (pg/ml) | N/A |
| --- | --- | --- |
|  | Expected [PFN-1] (pg/ml) | 78 |
|  | % Recovery | ***N/A*** |
| Negative + 625 pg/ml PFN-1 (n=5) | Mean [PFN-1] (pg/ml) | 294 |
|  | Expected [PFN-1] (pg/ml) | 312.5 |
|  | % Recovery | ***94%*** |
| Negative + 2500 pg/ml PFN-1 (n=5) | Mean [PFN-1] (pg/ml) | 1410 |
|  | Expected [PFN-1] (pg/ml) | 1250 |
|  | % Recovery | ***113%*** |

For PFN-1 (Cloud-Clone Corp., USCN Life Science Inc., SEC233Hu), spike experiments were performed by adding 50μl of a urine sample, negative for [PFN-1] to 50μl of three different standards of low, medium and high [PFN-1] in duplicates. (Low=195 pg/ml, medium=1563 pg/ml and high=12500pg/ml) The % recovery for the low and the medium standards were 74% and 84% respectively. The high standard gave an absorbance out of the range of the instrument (OD>3.0) Thus spiking [PFN-1] standards in urine yielded satisfactory results. According to the 4PL fit, % recovery could be calculated only for the medium and high standards. **(Table H)**

**Table H.** **Recovery study results of PFN-1**

| Negative + 195 pg/ml PFN-1 (n=2) | Mean [PFN-1] (pg/ml) | 100 |
| --- | --- | --- |
|  | Expected [PFN-1] (pg/ml) | 97.5 |
|  | % Recovery | ***103%*** |
| Negative + 1563 pg/ml PFN-1 (n=2) | Mean [PFN-1] (pg/ml) | *802* |
|  | Expected [PFN-1] (pg/ml) | 781.5 |
|  | % Recovery | ***103%*** |
| Negative + 12500 pg/ml PFN-1 (n=2) | Mean [PFN-1] (pg/ml) | *N/A* |
|  | Expected [PFN-1] (pg/ml) | 6250 |
|  | % Recovery | ***N/A*** |

For NIF-1 (CUSABIOBiotech CO. LTD, CSB-EL026683HU), spike experiments were performed by adding 50μl of a urine sample, negative for NIF-1 to 50μl of three different standards of low, medium and high [NIF-1] in duplicates. (Low=25 pg/ml, medium=100 pg/ml and high=800 pg/ml) The % Recovery of the low standard was 175%, of the medium 270% and of the high 335%, thus spiking [NIF-1] standards in urine did not yield a satisfactory result. According to the 4PL fit, the % recovery of the low [NIF-1] standard was 101%, of the medium [NIF-1] standard was 130% and of the high [NIF-1] standard was 281%. (**Table I**)

**Table I. Recovery study results of NIF-1**

| Negative + 25 pg/ml NIF-1 (n=2) | Mean [NIF-1] (pg/ml) | 12.6 |
| --- | --- | --- |
|  | Expected [NIF-1] (pg/ml) | 12.5 |
|  | % Recovery | ***101%*** |
| Negative + 100 pg/ml NIF-1 (n=2) | Mean [NIF-1] (pg/ml) | 65 |
|  | Expected [NIF-1] (pg/ml) | 50 |
|  | % Recovery | ***130%*** |
| Negative + 800 pg/ml NIF-1 (n=2) | Mean [NIF-1] (pg/ml) | 1125 |
|  | Expected [NIF-1] (pg/ml) | 400 |
|  | % Recovery | ***281%*** |

For NIF-1 (USCN Life, WUHAN EIAAB SCIENCE CO. LTD, E1019h), spike experiments were performed by adding 50μl of a urine sample, negative for NIF-1 to 50μl of three different standards of low, medium and high [NIF-1] in four (4) replicates. (Low=1.56 ng/ml, medium=6.25 ng/ml and high=25 ng/ml) The % Recovery was very low and not acceptable for all standards. (35% for the low standard, 56% for the medium standard and 39% for the high standard) Thus spiking [NIF-1] standards in urine did not yield a satisfactory result. According to the 4PL fit, the % recovery of the low [NIF-1] standard was 18%, of the medium [NIF-1] standard was 19% and of the high [NIF-1] standard was 14%. (**Table J**)

**Table J. Recovery study results of NIF-1**

| Negative + 1.56 ng/ml NIF-1 (n=4) | Mean [NIF-1] (ng/ml) | 0.14 |
| --- | --- | --- |
|  | Expected [NIF-1] (ng/ml) | 0.78 |
|  | % Recovery | ***18%*** |
| Negative + 6.25 ng/ml NIF-1 (n=4) | Mean [NIF-1] (ng/ml) | 0.59 |
|  | Expected [NIF-1] (ng/ml) | 3.13 |
|  | % Recovery | ***19%*** |
| Negative + 25 ng/ml NIF-1 (n=4) | Mean [NIF-1] (ng/ml) | 1.77 |
|  | Expected [NIF-1] (ng/ml) | 12.5 |
|  | % Recovery | ***14%*** |
