# Supplementary material for: Analytical Performance of ELISA Assays in Urine: One More Bottleneck towards Biomarker Validation and Clinical Implementation
Source: PLoS One. 2016 Feb 18;11(2):e0149471. doi: 10.1371/journal.pone.0149471 (PMC4758723; doi:10.1371/journal.pone.0149471)
Supplement: S9 File — (DOCX) [file pone.0149471.s009.docx]

**Table A. Inter-assay reproducibility results for SPARC, SLIT-2 and SURVIVIN**

| **Concentration** | **SPARC (ng/ml)** | |  | **SLIT-2 (pg/ml)** | |  | **SURVIVIN (pg/ml)** |
| --- | --- | --- | --- | --- | --- | --- | --- |
|  | **Low (n=2)** | **Medium (n=2)** | **High (n=2)** | **Low (n=2)** | **Medium (n=2)** | **High (n=2)** | **Low (n=2)** |
| **Mean** | **4.99** | **11.3** | **25.1** | **629** | **1157** | **2403** | **73** |
| **STDEV** | **1.44** | **0.99** | **8.4** | **272** | **391** | **256** | **30** |
| **CV** | **29%** | **9%** | **34%** | **43%** | **34%** | **11%** | **41%** |
