# Supplementary material for: Analytical Performance of ELISA Assays in Urine: One More Bottleneck towards Biomarker Validation and Clinical Implementation
Source: PLoS One. 2016 Feb 18;11(2):e0149471. doi: 10.1371/journal.pone.0149471 (PMC4758723; doi:10.1371/journal.pone.0149471)
Supplement: S10 File — (DOCX) [file pone.0149471.s010.docx]

**Table A. LOD and LOQ information for all the ELISA kits**

| **Protein** | **Company** | **Catalogue number** | **Limit of Detection (LOD)** | **Standard range** | **LOQ Low** | **LOQ High** |
| --- | --- | --- | --- | --- | --- | --- |
| **SPARC** | R&D Systems | DSP00 | 0.044 ng/ml | 1.56-50 ng/ml | 1.60 ng/ml | 50 ng/ml |
| **SLIT-2** | Cloud Clone Corp. | SEA672Hu | < 26 pg/ml | 78-5000 pg/ml | 37.2 pg/ml | 4992 pg/ml |
| **H2B** | US Biological Life Sciences | 25705 | < 1.28 ng/ml | 3.12-200 ng/ml | 0.83 ng/ml | 197.6 ng/ml |
|  | Cloud Clone Corp. | SEA356Hu | < 1.33 ng/ml | 3.12-200 ng/ml | 3.12 ng/ml | 200.5 ng/ml |
| **SURVIVIN** | Enzo Life Sciences | ADI-900-111 | 4 pg/ml | 31.25-500 pg/ml | 30.0 pg/ml | 500.8 pg/ml |
|  | R&D Systems | DSV00 | 4.44 pg/ml | 31.2-2000 pg/ml | 45.7 pg/ml | 2002 pg/ml |
| **PFN-1** | USCN LIFE | E2122h | 33 pg/ml | 78-5000 pg/ml | 69.3 pg/ml | 5080 pg/ml |
|  | US Biological Life Sciences | 27613 | 28 pg/ml | 78-5000 pg/ml | 66.0 pg/ml | 4996 pg/ml |
|  | Cloud Clone Corp. | SEC233Hu | 28 pg/ml | 78.1-5000 pg/ml | 90.1 pg/ml | 5204 pg/ml |
| **NIF-1** | CUSABIO | EL026683HU | 6.25 pg/ml | 25-1600 pg/ml | 19.1 pg/ml | 1561 pg/ml |
|  | USCN LIFE | E1019h | 0.1 ng/ml | 0.31-20 ng/ml | 0.30 ng/ml | 14.56 ng/ml |
| **Proteinase 3** | CUSABIO | E13058h | 0.195 ng/ml | 0.78-50 ng/ml | 0.71 ng/ml | 49.9 ng/ml |
