# Supplementary material for: Analytical Performance of ELISA Assays in Urine: One More Bottleneck towards Biomarker Validation and Clinical Implementation
Source: PLoS One. 2016 Feb 18;11(2):e0149471. doi: 10.1371/journal.pone.0149471 (PMC4758723; doi:10.1371/journal.pone.0149471)
Supplement: S11 File — (DOCX) [file pone.0149471.s011.docx]

**Table A.** **Chi-square test results of SPARC and hematuria**

|  |  |  | **SPARC**  **(Chi-square value=2,060 pvalue=0,151)** | | **Total** |
| --- | --- | --- | --- | --- | --- |
|  |  |  | **negative** | **positive** |  |
| **Hematuria** | **Absent** | **Count** | 41 | 8 | 49 |
|  |  | **% within Hematuria** | 83,7% | 16,3% | 100,0% |
|  |  | **% within SPARC** | 66,1% | 47,1% | 62,0% |
|  |  | **% of Total** | 51,9% | 10,1% | 62,0% |
|  | **Present** | **Count** | 21 | 9 | 30 |
|  |  | **% within Hematuria** | 70,0% | 30,0% | 100,0% |
|  |  | **% within SPARC** | 33,9% | 52,9% | 38,0% |
|  |  | **% of Total** | 26,6% | 11,4% | 38,0% |
| **Total** | | **Count** | 62 | 17 | 79 |
|  |  | **% within Hematuria** | 78,5% | 21,5% | 100,0% |
|  |  | **% within SPARC** | 100,0% | 100,0% | 100,0% |
|  |  | **% of Total** | 78,5% | 21,5% | 100,0% |
