# Supplementary material for: Analytical Performance of ELISA Assays in Urine: One More Bottleneck towards Biomarker Validation and Clinical Implementation
Source: PLoS One. 2016 Feb 18;11(2):e0149471. doi: 10.1371/journal.pone.0149471 (PMC4758723; doi:10.1371/journal.pone.0149471)
Supplement: S12 File — (DOCX) [file pone.0149471.s012.docx]

**Table A.** **Chi-square test results of SLIT-2 and hematuria**

|  |  |  | **SLIT-2**  **(Chi-square value=0,081 pvalue=0,776)** | | **Total** |
| --- | --- | --- | --- | --- | --- |
|  |  |  | **Negative** | **Positive** |  |
| **Hematuria** | **Absent** | **Count** | 7 | 99 | 106 |
|  |  | **% within Hematuria** | 6,6% | 93,4% | 100,0% |
|  |  | **% within SLIT2** | 63,6% | 67,8% | 67,5% |
|  |  | **% of Total** | 4,5% | 63,1% | 67,5% |
|  | **Present** | **Count** | 4 | 47 | 51 |
|  |  | **% within Hematuria** | 7,8% | 92,2% | 100,0% |
|  |  | **% within SLIT2** | 36,4% | 32,2% | 32,5% |
|  |  | **% of Total** | 2,5% | 29,9% | 32,5% |
| **Total** | | **Count** | 11 | 146 | 157 |
|  |  | **% within Hematuria** | 7,0% | 93,0% | 100,0% |
|  |  | **% within SLIT2** | 100,0% | 100,0% | 100,0% |
|  |  | **% of Total** | 7,0% | 93,0% | 100,0% |
