# Supplementary material for: Analytical Performance of ELISA Assays in Urine: One More Bottleneck towards Biomarker Validation and Clinical Implementation
Source: PLoS One. 2016 Feb 18;11(2):e0149471. doi: 10.1371/journal.pone.0149471 (PMC4758723; doi:10.1371/journal.pone.0149471)
Supplement: S13 File — (DOCX) [file pone.0149471.s013.docx]

**Table A.** **Chi-square test results of SURVIVIN and hematuria**

|  |  |  | **SURVIVIN**  **(Chi-square value=7,935**  **Pvalue=0,005)** | | **Total** |
| --- | --- | --- | --- | --- | --- |
|  |  |  | **Negative** | **Positive** |  |
| **Hematuria** | **Absent** | **Count** | 81 | 4 | 85 |
|  |  | **% within Hematuria** | 95,3% | 4,7% | 100,0% |
|  |  | **% within SURVIVIN** | 69,8% | 30,8% | 65,9% |
|  |  | **% of Total** | 62,8% | 3,1% | 65,9% |
|  | **Present** | **Count** | 35 | 9 | 44 |
|  |  | **% within Hematuria** | 79,5% | 20,5% | 100,0% |
|  |  | **% within SURVIVIN** | 30,2% | 69,2% | 34,1% |
|  |  | **% of Total** | 27,1% | 7,0% | 34,1% |
| **Total** | | **Count** | 116 | 13 | 129 |
|  |  | **% within Hematuria** | 89,9% | 10,1% | 100,0% |
|  |  | **% within SURVIVIN** | 100,0% | 100,0% | 100,0% |
|  |  | **% of Total** | 89,9% | 10,1% | 100,0% |
